# Pharmacological potentials of lycopene against aging and aging‐related disorders: A review

**DOI:** 10.1002/fsn3.3523

**Published:** 2023-06-27

**Authors:** Mehedy Hasan Abir, A. G. M. Sofi Uddin Mahamud, Sadia Haque Tonny, Mithila Saha Anu, K. H. Sabbir Hossain, Ismam Ahmed Protic, Md Shihab Uddine Khan, Artho Baroi, Akhi Moni, Md Jamal Uddin

**Affiliations:** ^1^ ABEx Bio‐Research Center Dhaka Bangladesh; ^2^ Faculty of Food Science and Technology Chattogram Veterinary and Animal Sciences University Chattogram Bangladesh; ^3^ Department of Food Safety and Regulatory Science Chung‐Ang University Anseong‐si Gyeonggi‐do Republic of Korea; ^4^ Faculty of Agriculture Bangladesh Agricultural University Mymensingh Bangladesh; ^5^ Department of Fisheries Biology and Genetics Faculty of Fisheries, Bangladesh Agricultural University Mymensingh Bangladesh; ^6^ Department of Plant Pathology Faculty of Agriculture, Bangladesh Agricultural University Mymensingh Bangladesh; ^7^ Department of Crop Botany Faculty of Agriculture, Bangladesh Agricultural University Mymensingh Bangladesh

**Keywords:** anti‐aging, calorie restriction, chronic disease, inflammation, lycopene, oxidative stress

## Abstract

Aging and aging‐related chronic disorders are one of the principal causes of death worldwide. The prevalence of these disorders is increasing gradually and globally. Considering this unwavering acceleration of the global burden, seeking alternatives to traditional medication to prevent the risk of aging disorders is needed. Among them, lycopene, a carotenoid, is abundant in many fruits and vegetables, including tomatoes, grapefruits, and watermelons, and it has a unique chemical structure to be a potent antioxidant compound. This nutraceutical also possesses several anti‐aging actions, including combating aging biomarkers and ameliorating several chronic disorders. However, no systematic evaluation has yet been carried out that can comprehensively elucidate the effectiveness of lycopene in halting the course of aging and the emergence of chronic diseases linked to aging. This review, therefore, incorporates previous pre‐clinical, clinical, and epidemiological studies on lycopene to understand its potency in treating aging disorders and its role as a mimic of caloric restriction. Lycopene‐rich foods are found to prevent or attenuate aging disorders in various research. Based on the evidence, this review suggests the clinical application of lycopene to improve human health and alleviate the prevalence of aging and aging disorders.

## Graphical abstract

Lycopene can be a prospective pharmaceutical agent for managing aging‐related chronic diseases because it can delay aging by attenuating aging biomarkers or acting as a calorie restriction regimen that can prevent metabolic disturbance and chronic disease development.

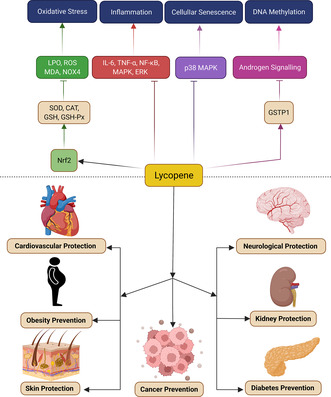

## 1 INTRODUCTION

As life expectancies increase, the number of aging and aging‐related complexities is also accelerating gradually among the global population. It is estimated that older adults over 60 years old will become 22% from 11% within 2000–2050, and the number might become 2 billion from 605 million (MacNee et al., 2014). Aging is the most prevailing risk factor for non‐communicable chronic aging‐related disorders such as cardiovascular diseases, cancers, diabetes mellitus, neurological disorders, and kidney diseases, leading to about 100,000 deaths per day worldwide. These aging‐related diseases accounted for 29.5 million deaths (72% of total death) among 54.7 million deaths in 2016 (Harris, 2019). Epidemiological studies show that a healthy diet containing fruits and vegetables correlates with a reduced progression of aging or various aging‐related disorders (Arif et al., 2022; Zhou et al., 2022). Though several medications are available for these diseases (Li et al., 2021), most of them manifest side effects upon implementation, and they might also become futile if the disease gets uncontrollable. Therefore, nutraceutical‐based treatment is now getting attention in the aging and chronic diseases research area of interest.

A carotenoid compound‐lycopene is abundant in tomatoes and many tomato products, and small amount IF found in some other fruits, including apricot, watermelon, pink guava, and others (Rao & Rao, 2018); it has become of great interest to the public health research. Though red‐ and orange‐colored fruits and vegetables are the primary sources of this lipid‐soluble pigment, some green‐colored fruits and vegetables, for example, asparagus and parsley, are also modest sources of this compound (Hedayati et al., 2019; Yin et al., 2019). Lycopene is a tetra‐terpene constituent that comprises 8‐isoprene hydrocarbons and 11 linearly double bonds. It is also known as a non‐provitamin‐A carotenoid (Pennathur et al., 2010; Yin et al., 2019).

A recent study demonstrated that lycopene concentration is the highest among all naturally found carotenoids in the serum, blood, and organ tissues (Saini et al., 2020). Nonetheless, lycopene is one of the most potent anti‐oxidant, which can inhibit the generation of reactive oxygen species (ROS) and remove singlet oxygen double compared to β‐carotene and 10 times more than α‐tocopherol (Przybylska, 2020). The functional role of lycopene is not limited to antioxidant activity; it has several other health benefits that brought it to the focus of the public health research area of interest (Joshi et al., 2020). A review suggests that lycopene and the consumption of foods high in it may reduce the chance of developing age‐related diseases, such as cancer and cardiovascular disease (CVD) (Story et al., 2010). As the increasing bioavailability of lycopene can combat aging and age‐related disorders, it should be supplemented in increased amounts to upregulate its circulation in blood and serum (Ellis et al., 2019; Petyaev, 2016). The functional activities of lycopene against aging disorders may contribute to lifespan expansion and healthy aging in humans. However, no systematic review has been performed to comprehensively elucidate the efficacy of lycopene in preventing the aging process and the manifestation of aging‐related chronic diseases. Instead, most studies individually discussed the biofunctional roles of lycopene against particular aging biomarkers or aging‐related chronic comorbidities.

Considering these, the current study comprehensively reviews the mechanisms that underpin the anti‐aging features of lycopene to elucidate its role in aging biomarkers and aging‐related disorders, which is crucial for designing lycopene‐based therapeutics for clinical application. In addition, the review provides some speculative opinions on the limitations, possible solutions, and future research directions to establish the nutraceutical as a potent anti‐aging drug.

## 2 METHODS

### 2.1 Research question and definitions

The patient‐intervention‐comparison‐outcome‐study design (PICOS) model (Schardt et al., 2007) was followed before searching the literature to assure a few specific research questions and objectives of the review study. This study primarily screened out the existing evidence regarding lycopene administration in human and non‐human models to underpin the prospective applications of lycopene in pharmaceutical industries for developing anti‐aging drugs. The inclusion and exclusion criteria followed before screening the literature are given in Table 1. From the PICOS, the specific review questions were developed:

- What is the present state of evidence for the efficacy of lycopene administration in the prevention of aging and the progression of age‐related chronic diseases?
- Can lycopene act as a calorie restriction mimic in reducing the onset of aging?
- What are the limits of lycopene administration in the management of aging problems?

**TABLE 1:** A list of inclusion and exclusion criteria for the review.

Parameters	Inclusion	Exclusion
Publication year	January 2001–October 31, 2021	Prior to 2001
Study type	Peer‐reviewed, published original research articles Human studies Animal studies Cell studies	Systematic reviews, meta‐analyses, commentaries, and non‐peer‐reviewed articles Dissertations and unpublished works
Age	Adults aged 18 years or older	Pediatric population (ages less than 18 years)
Gender	Male Female	Excluded unusual gender identities, including transgender, non‐binary, agender, gender dysphoria
Types of aging biomarker	Oxidative stress Inflammation DNA damage DNA methylation Telomere length shortening Cell senescence	Epigenetic markers, such as histone loss, histone variants, modification Transcriptomic biomarkers Loss of proteostasis Mitochondrial dysfunction
Types of aging‐related disease	Obesity Diabetes Cancer Cardiovascular diseases Skin aging Kidney disorders Neurological disorders	Hair and teeth loss Macular degeneration Musculoskeletal disorders Respiratory disease Infertility
Supplement type	Fresh raw tomato Purified lycopene Lycopene‐based medicine	Tomato ketchup Tomato sauce Tomato‐containing fast food items, such as Pizza

### 2.2 Search methods

**FIGURE 1:**
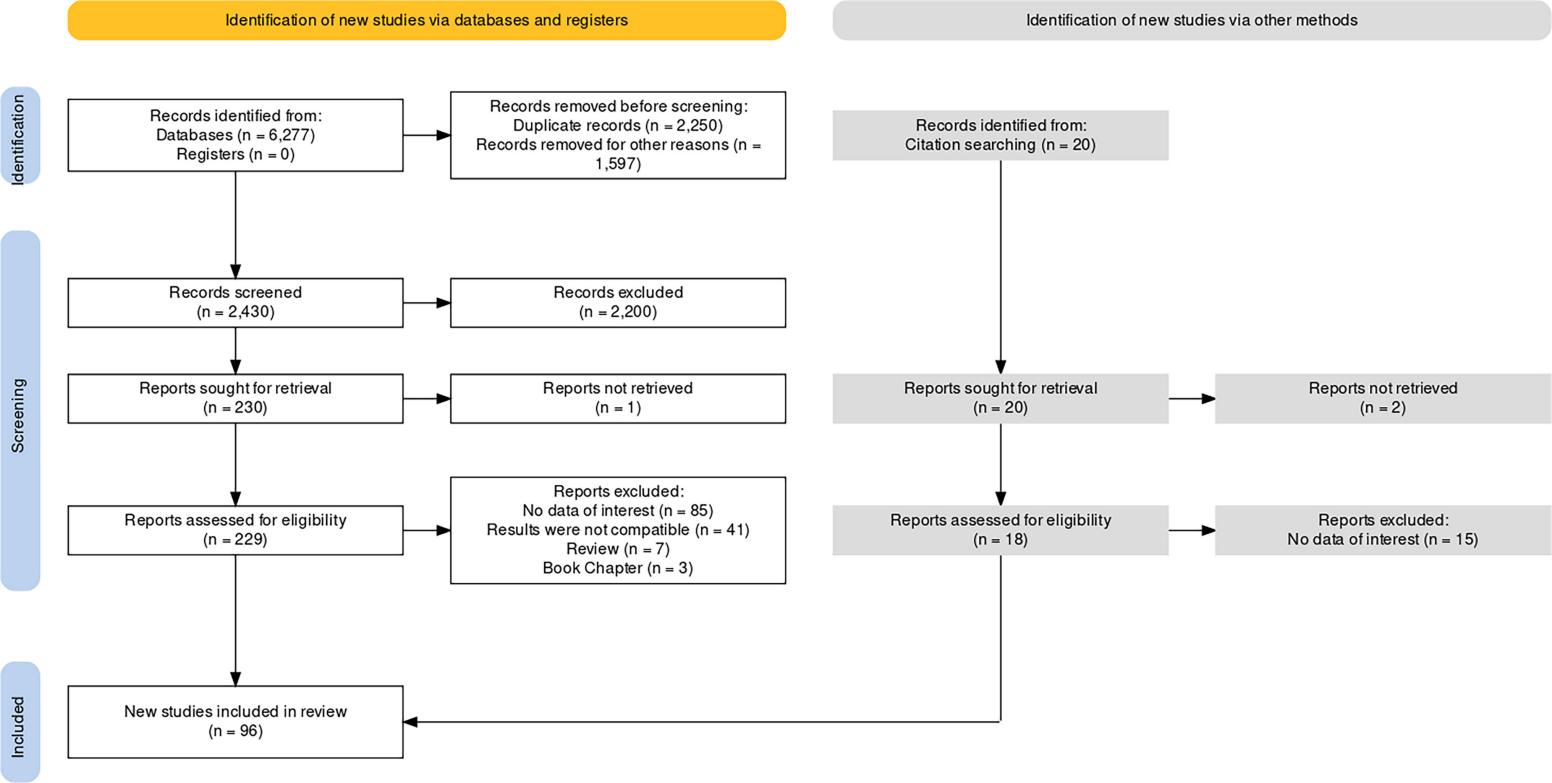
PRISMA 2020 protocol of the systematic review.

This review article adhered to the standard systematic review procedures established by the preferred reporting items for systematic reviews and meta‐analyses (PRISMA) (Page et al., 2021). The procedural guidelines of PRISMA 2020 are shown in Figure 1 (Haddaway et al., 2022), where the following procedural standards were followed: database searching to identify potentially relevant articles, relevance evaluation, quality assessment, and data extraction. We performed literature searching of original research and review articles of the last 20 years (from 2001 to October 2021) using PubMed, Scopus, and Google Scholar on the effects of lycopene against various aging biomarkers and age‐related chronic disorders. Firstly, we searched using various keywords, including lycopene, aging, oxidative stress, inflammation, DNA alterations, telomere length, and cellular senescence. Later, we searched the literature on the effects of lycopene against various aging‐related chronic complications by using several keywords, including obesity, diabetes, cancer, CVD, skin aging, kidney disorders, neurological disorders, pre‐clinical‐clinical trials, and drug development.

## 3 CHEMISTRY AND BIOCHEMISTRY OF LYCOPENE

Lycopene is one of the crucial compounds of the carotenoid pigment family. Carotenoids can manifest several colors in a plant, mainly because of lutein and lycopene groups. Carotenoid pigments are also pivotal in metabolizing vitamin A and several other metabolites. Carotenoids that comprise only hydrogen and carbon in the structure are called hydrocarbon carotenoids, whereas oxygen, hydrogen, and carbon‐comprising carotenoids are called xanthophyll carotenoids (Story et al., 2010).

The chemical and biochemical properties of lycopene hold great importance because it can act as a therapeutic agent and manifest several health benefits when transformed into an active form. Its structure, chemistry, and biochemistry manifest the way of its activity and mechanism of the reaction. Lycopene is a non‐cyclic carotenoid because of its 11 linear double bonds in the structure, and its chemical formula is C_40_H_56_ (Bunghez et al., 2011). It does not assist in vitamin‐A metabolism because it lacks ring structures. However, lycopene shows sensitivity under adverse abiotic circumstances, such as higher temperatures, pH, and lightly stressed conditions (Srivastava & Srivastava, 2015). These factors cause significant changes in its structure. As a result, lycopene's *trans*‐state is changed to a *cis*‐state, which affects its bioavailability in humans and thus manifests substantive health benefits in the body. The *cis‐*state of lycopene is more stable than the *trans*‐state and contains a lower melting point, greater oil solubility, and lesser crystallization occurrence. These characteristics make the *cis* form of lycopene more biologically available in humans (Shi & Le Maguer, 2000; Srivastava & Srivastava, 2015). Though the *cis‐*state of lycopene is the most prevalent in the human body, its isomerization into a *trans‐*state can occur in the stomach, liver, and intestines (Richelle et al., 2010; Teodoro et al., 2009). Walfisch et al. (2003) observed no changes in isomer distribution in serum and tissues after supplementing tomato lycopene oleoresin with highly concentrated all‐*trans* isomer and found more than 90% lycopene in the all‐*trans* form. Its absorption in the intestine is mainly expedited by two scavenger receptors named CD36 and B1 (Moussa et al., 2011, 2008), and partial metabolism occurs in the enterocytes (Imran et al., 2020). However, lycopene's oxidizing and isomerizing characteristics are two important reasons for altering its therapeutic potential because they can affect nutrient contents and color‐imparting properties of lycopene. Moreover, numerous thermal and non‐thermal operations have been proven to damage the structures of lycopene (Martínez‐Hernández et al., 2016).

## 4 POTENTIALS OF LYCOPENE AGAINST AGING BIOMARKERS AND AGING‐RELATED DISORDERS

**FIGURE 2:**
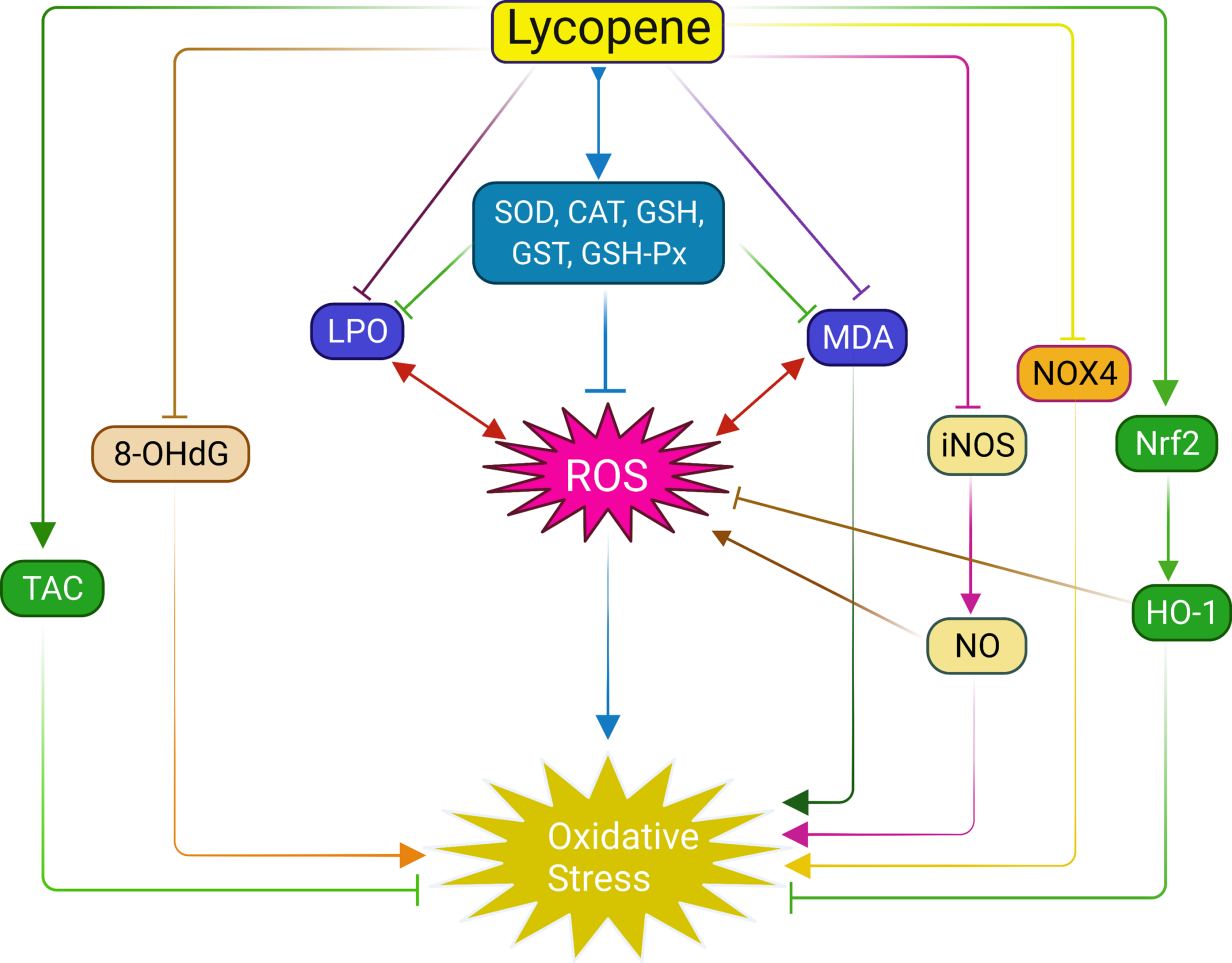
Anti‐oxidative mechanism of lycopene. Lycopene inhibits ROS generation and subsequent oxidative stress by inducing antioxidant enzymes (SOD, CAT, GSH, GSH‐Px, and GST) and limiting MDA level and lipid peroxidation (LPO). Lycopene also prevents ROS release by upregulating Nrf2‐mediated HO‐1 levels and inhibiting iNOS‐activated NO generation. In addition, lycopene prevents oxidative stress through upregulating total antioxidant capacity (TAC) and direct inhibition of 8‐OHdG, NOX4.

**FIGURE 3:**
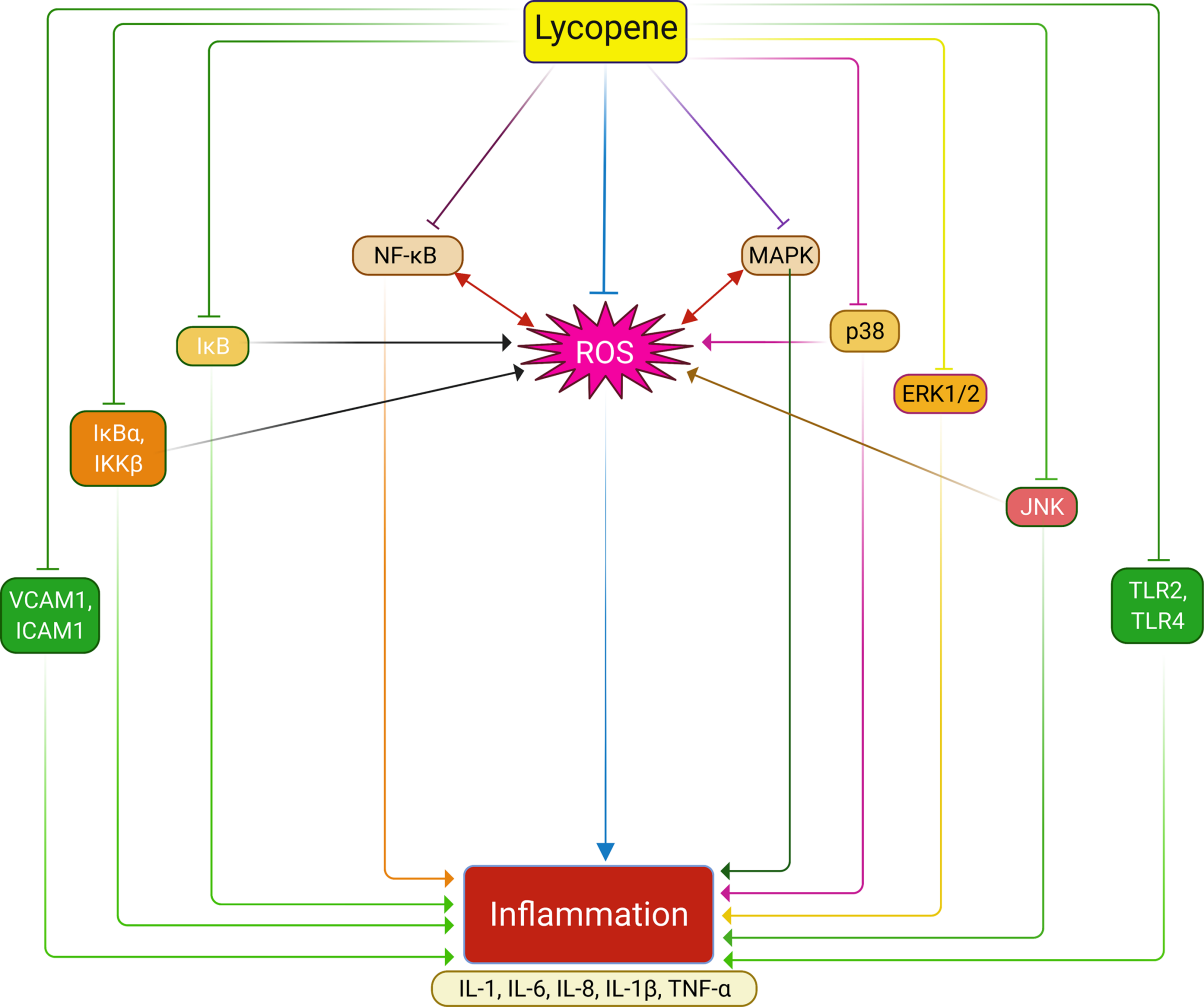
Anti‐inflammatory mechanism of lycopene. Lycopene inhibits inflammation by reducing ROS generation and inflammatory mediators. Primarily, lycopene inhibits ROS production, which plays an interchangeable role in proinflammatory cytokines, including IL‐1, IL‐6, IL‐8, IL‐1β, and TNF‐α release. In addition, lycopene inhibits the MAPK pathway and its isomers p38, ERK1/2, and JNK pathways, subsequently leading to ROS generation and proinflammatory cytokines releases. Similarly, lycopene suppressed phosphorylation of IκB and its phosphorylates IκBα and IKKβ and subsequent activation of the NF‐κB pathway. In addition, lycopene prevents inflammation by inhibiting toll‐like receptors TLR2 and TLR4 and endothelial adhesion molecules VCAM1 and ICAM‐1.

**FIGURE 4:**
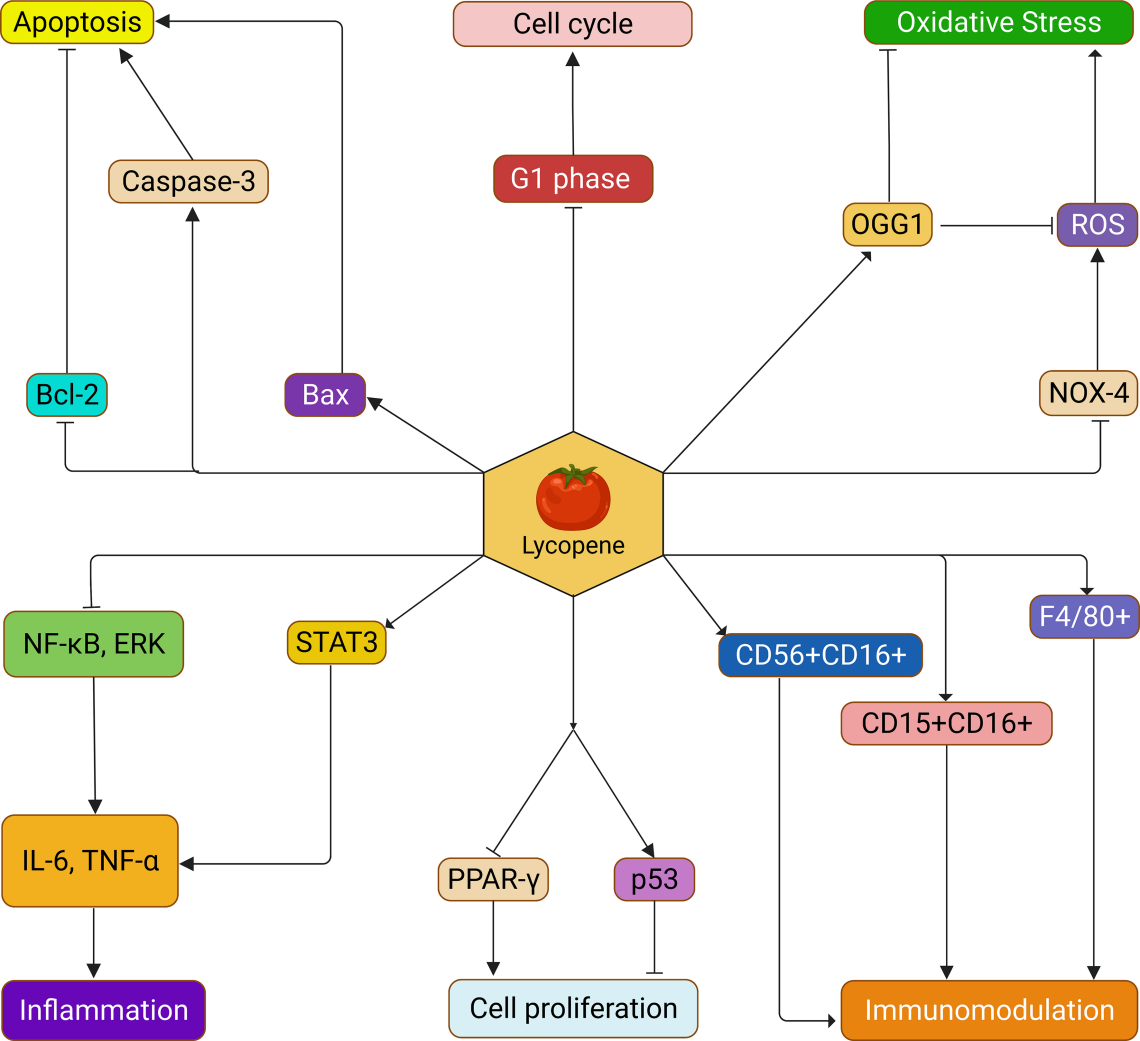
Preventive actions of lycopene against cancer‐related mechanisms. Lycopene exhibits different modes of action against cancer, such as elimination of oxidative stress, inflammation, apoptosis, regulation of cell cycle and cell proliferation, and immunomodulation. Lycopene inhibits oxidative stress‐mediated cancer development inducing OGG1 and inhibiting NOX4‐mediated ROS generation. Lycopene also prevents inflammatory response mediated cancer development by inhibiting STAT3, NF‐κB, ERK pathway, and IL‐6 and TNF‐α release. In addition, lycopene regulates apoptosis by downregulating Bcl2 and inducing Bax protein and cleaved Caspase‐3 levels. Furthermore, lycopene arrests the G1 phase and the cell cycle, inhibits PPAR‐γ, and induces p53 protein to regulate cell proliferation. Lycopene also shows immunomodulatory actions and prevents cancer development by upregulating NK immunity cell marker CD56+CD16+, macrophage immunity cell marker F4/80+, and neutrophil immunity cell marker CD15+CD16+.

Aging is a natural degradation process, which is the cumulative outcome of various detrimental dynamic alternations or damages in molecular pathways and cellular parts. The aging process is characterized by changes in biochemical composition in tissues, gradual decreases in physiological capacity and responding ability to environmental changes, enhanced susceptibility and vulnerability to various non‐communicable diseases, and increased mortality (MacNee et al., 2014; Troen, 2003). Several biomarkers are associated with aging, such as DNA damage, DNA methylation, telomere length shortening, cell senescence, oxidative stress, inflammation, and many other biomarkers (Xia et al., 2017). Aging also acts as a risk factor for several chronic diseases, including cardiovascular diseases, kidney disorders, neurological disorders, cancer, diabetes, and obesity, and accelerates their severity and morbidity (Prasad et al., 2012). Numerous studies have demonstrated that lycopene has significant effects on aging biomarkers, prevents the aging process, and minimizes the severity of age‐related chronic diseases, which have been described in the following sections, summarized in Tables 2, 3, 4, and illustrated in Figures 2, 3, 4.

**TABLE 2:** Effects of lycopene against aging biomarkers.

Study type	Study subjects	Dose	Duration	Major functions	Molecular biomarkers	References
Animal studies	HgCl_2_‐induced Wistar rats (40 rats)	5 mL/kg body weight (oral gavage and injection)	2 days	Antioxidation	MDA, ROS↓ GSH‐Px, SOD, GSH↑	Yang et al. (2011)
	Male albino rats (24 adult rats)	10 mg/kg. BW (orally)	5 weeks	Antioxidation	MDA, LPO, Total nitrate/nitrite↓ GSH, SOD, TAC↑	Mansour and Tawfik (2012)
	Cisplatin‐induced male Wistar rats (28 rats)	6 mg/kg. BW/day (Single‐injection)	10 days	Antioxidation, anti‐inflammation	NF‐κB p65↓ Nrf2, and HO‐1↑ GSH, CAT, GPx, and SOD↑	Sahin et al. (2010)
	Colistin‐induced female Kunming mice	5 or 20 mg/kg. BW/day (orally)	7 days	Antioxidation	LPO↓ HO‐1↑ Nrf2, HO‐1 mRNA↑ GSH, CAT, SOD↑ NF‐κB mRNA↓	Dai et al. (2015)
	Croton oil‐induced male Kunming mice	0.5 g/kg (oral gavage)	4 days	Anti‐inflammation	Edema rate↓	Yaping et al. (2003)
	Streptozotocin‐induced diabetic Wistar rats	1, 2 and 4 mg/kg	10 weeks	Antioxidation, anti‐inflammation	Oxidative stress, NO↓ TNF‐α production↓	Kuhad, Sethi, and Chopra (2008)
	Hyperhomocysteinemic Sprague–Dawley rats (50 rats)	10, 15, and 20 mg/kg	12 weeks	Anti‐inflammation	VCAM‐1, MCP‐1, IL‐8↓	Liu et al. (2007)
	Alcohol‐fed Fischer 344 rats (60 rats)	1.1, 3.3 mg/kg. BW/day	11 weeks	Anti‐inflammation	TNF‐α mRNA, hepatic inflammatory foci↑	Veeramachaneni et al. (2008)
	Ovalbumin (OVA)‐induced BALB/c mice	8 or 16 mg/kg BW/day in 200 μL (IP injection)	3 days	Anti‐inflammation	IFN‐γ and T‐bet mRNA↑ IL‐4 mRNA↓	Lee et al. (2008)
	Young male and female rats (48 rats/sex)	0, 267, 534, 1068 mg/kg. BW	30 days	Antioxidation	CAT, SOD & GSH‐Px↑ MDA, ROS formation↓	Hu et al. (2013)
	28 male Wistar‐Albino male rats	10 mg/kg. BW/day	28 days	Antioxidation	Blood glucose and HbA1c↓ Oxidative DNA damage↓ 8‐OHdG↓	Karahan et al. (2018)
Clinical trials	Prostate cancer patients (32 men)	30 mg/day	3 weeks	Antioxidation	Oxidative DNA damage↓	Chen et al. (2001)
	Type 2 diabetic patients (57 patients)	500 mL/day	4 weeks	Antioxidation	Plasma lycopene level (3‐fold)↑ LDL resistance to oxidation↑	Upritchard et al. (2000)
	Healthy, normolipidemic female volunteers (12 females)	8 mg/day	3 weeks	Antioxidation	Plasma lycopene level↑ 8 iso‐PGF2α in urine↓ LDL susceptibility to oxidation↓	Visioli et al. (2003)
	Well‐nourished, healthy elderly persons (33 female and 20 male)	330 mL/day tomato or, 47.1 mg lycopene	8 weeks	Immunomodulation	Plasma lycopene level↑ TNF‐α, and IL‐4↑ IL‐2↓	Watzl et al. (2000)
	Healthy young volunteers (26 individuals)	5.7 mg	26 days	Anti‐inflammation	TNF‐α↓	Riso et al. (2006)
	Healthy, non‐smoking post‐menopausal women (37 women)	4 mg/day (mixed supplementation) and 12 mg/day (individually)	56 days	Antioxidation	Oxidative DNA damage↓	Zhao et al. (2006)
Cell level study	Lipopolysaccharide (LPS)‐mediated (RAW 264.7) Murine macrophages	0.5, 1, 2 μM	24 h	Anti‐inflammation	IL‐6 and IL‐1β mRNA↓ JNK phosphorylation↓	Marcotorchino et al. (2012)
	Lipopolysaccharide (LPS)‐mediated (RAW 264.7) macrophages	1–10 μM	24 h	Antioxidation, anti‐inflammation	mRNA of iNOS, NO↓ IL‐6↓ p38, ERK1/2, IκB phosphorylation↓ NF‐κB translocation↓	Feng et al. (2010)
	THP‐1 (human monocytic cell line)	0.5–2 μM	6 h	Antioxidation, anti‐inflammation	IL‐8↓ ROS and NOX‐4↓ NF‐κB DNA binding, NF‐κB/p65 nuclear translocation↓ IKKα and IκBα phosphorylation↓ ERK1/2, JNK, p38 MAPK phosphorylation↓	Simone et al. (2011)
		0.5–2 μM	24 h	Antioxidation, anti‐inflammation	ROS and 8‐OHdG formation↓ NOX‐4 and NADPH oxidase↓ Hsp70, Hsp90 expressions↓ p38, ERK, JNK, MAPK phosphorylation↓	Palozza et al. (2010)
	3 T3‐L1 (murine pre‐adipocytes)	0.5, 1, or 2 μM	24 h	Anti‐inflammation	mRNA expression of IL‐6, MCP‐1, IL‐1β, RANTES, CXCL1, CXCL10, SAA3, and haptoglobin↓	Gouranton et al. (2011)
	LPS‐induced Human umbilical vein endothelial cells (HUVECs)	0–20 μM	6 h	Anti‐inflammation	HMGB1, HMGB1‐mediated TNF‐α and sPLA2‐IIA↓ VCAM‐1, ICAM‐1, E‐selectin↓ TLR‐2, TLR‐4, and receptors of RAGE↓	Lee et al. (2012)

*Note*: ↑ = increase; ↓ = decrease/inhibit; → = normal/no change.

Abbreviations: 8‐OHdG, 8‐hydroxy‐2′‐deoxyguanosine; CAT, catalase; CXCL, chemokine (C‐X‐C motif) ligand; ERK, extracellular signal‐regulated kinases; GSH, glutathione; GSH‐Px, glutathione peroxidase; HMGB1, high mobility group box 1; HO‐1, heme oxygenase‐1; ICAM‐1, intercellular adhesion molecule‐1; IFN‐α, interferon type I; IKKα, IκB kinase α; IL, interleukin; iNOS, inducible nitric oxide synthase; JNK, c‐Jun N‐terminal kinases; LPO, lipid peroxidation; MAPK, mitogen‐activated protein kinases; MCP‐1, monocyte chemoattractant protein‐1; MDA, malondialdehyde; NF‐κB, nuclear factor kappa B; NO, nitric oxide; NOX4, NADPH oxidase 4; Nrf2, nuclear factor erythroid 2–related factor 2; PGF2α, prostaglandin F2α; RANTES, regulated upon activation, normal T cell expressed and presumably secreted; SOD, superoxide dismutase; sPLA2‐IIA, group II secretory phospholipase A; TLR, toll‐like receptor; TNF‐α, tumor necrosis factor‐alpha; VCAM‐1, vascular cell adhesion molecule‐1.

**TABLE 3:** Pre‐clinical evidence on protective actions of lycopene against age‐related disorders.

Properties	Study type	Study subjects	Dose	Duration	Major outcomes	Molecular markers	References
Obesity Prevention	Animal studies	Male Swiss albino mice	5, 10 mg/kg	12 weeks	Prevented weight gain and adiposity, improved adipose tissue fat mobilization, and reduced insulin resistance	Serum TG level, systemic adiposity↓ Insulin sensitivity, glucose clearance↑ Liver glucose/lipid metabolism↑	Singh et al. (2016)
		Male C57BL/6J mice	0.03%	10 weeks	Improved lipid metabolism, prevented insulin resistance, inflammation, and obesity	Weight gain↓ adipocytes size↓ *Fas*, *Acaca*, *Pparγ*, *Srebp1c* and *Pparg*↓ *Ucps*, *Ebf2*, and *Pgc1α*, *Prdm16*↑ *Cox5b*, *Cox8b*, *Cycs*, *Sirt1*, and *CoxII*↑ PPARɑ, SIRT1, *Cpt1ɑ*, *Ucp1*, *Cidea*↑ *Fgf21*↓ *Atg7*, *Atg14*, *Lc3*, *P62*, *Beclin*↑ *leptin*↓, *Glut1*, *Glut4*↑ IL‐1β, IL‐6, TNF‐α↓ *Zo‐1*, *Claudin‐1*, and *Occludin*↑	Wang et al. (2019)
		Male C57BL/6J mice	10 mg/kg	12 weeks	Prevented weight gain, and obesity‐associated pathologies	Adipose tissues mobilization↑ TG, 8‐iso‐PGF2α, and NEFA concentrations↓ HOMA‐IR index↓ Adipocyte hypertrophy↓ PPARγ mRNA (*ap2*, *Cd36*, *Lpl*)↓ *Fasn* and *Acaca*↓	Fenni et al. (2017)
		Male Wistar rats	25, 50 mg/kg. BW/day	3 months	Prevented obesity and associated complications	Weight gain, liver weight↓ Cholesterol, TG, Apo‐B, LDL‐c↓ HDL‐c↑, hepatic PPAR‐γ↑ SOD, CAT, GPx, GR, and GSH↑ MDA, NO↓ IL‐1β, TNF‐α, and MPO↓ Lactate dehydrogenase, creatine kinase↓ TGF‐β1, α‐SMA↓	Albrahim and Alonazi (2021)
		Male Wistar rats	0.01%	12 weeks	Attenuated metabolic syndrome and prevented the risk of obesity and CVD	→Abdominal fat, BMI, LVH, and liver index ROS↓ Hepatocytes and adipocytes size↓ Lipid accumulation in the liver↓	Ferreira‐Santos et al. (2020)
Diabetes	Animal studies	Male albino Wistar rats	1, 2 and 4 mg/kg. BW	8 weeks	Attenuated diabetic neuropathy	Plasma glucose, body weight↓ %MPE↑ TNF‐α and NO release↓	Kuhad, Sharma, and Chopra (2008)
		Male albino Wistar rats	10 or 20 mg/kg. BW	10 weeks	Improved glycolipid metabolism and prevented oxidative stress and the risk of T2DM	SOD and GSH‐Px↑, MDA↓ serum TG, TC, and LDL↓ Index of GHb and Gly‐LDL↓ FBG, HOMA‐IR↓ Serum insulin↑	Yin et al. (2019)
		Streptozotocin‐induced diabetic rats	45 mg/kg. BW	35 days	Improved glycemic index, prevented glycoxidative stress and diabetic‐associated complexities	Glucose tolerance and lipid profile↑ PON‐1 activity↑ Post‐prandial hyperglycemia, plasma cholesterol and ages↓ LPO↓, SOD↑	Figueiredo et al. (2020)
		Sprague–Dawley rats	10 mg/kg. BW/day	30 days	Prevented the risk of diabetes mellitus	Pancreatic vacuolization↓ Blood and urine glucose levels↓ Serum insulin levels↑	Ozmen et al. (2016)
Skin aging protection	Animal studies	Swiss albino female mice	5% or 10%	9 weeks	Protected from photoaging	TBARS↓, Collagen↑ CAT, GSH↑ →Epidermal thickness	Shah and Mahajan (2014)
		Outbred SKH‐1 hairless mice	22.1 or 60.9 mg/kg feed	35 weeks	Protected from UV radiation‐induced keratinocyte carcinomas	Tumor number↓, lycopene level in skin↑ Tomatidine presence↑	Cooperstone et al. (2017)
Cancer prevention	Animal studies	Balb/c male nude mice	0, 1, 5, or 10 mg/kg. BW	7 days	Suppressed the inflammatory response and prevented prostate cancer progression	Tumor volume↓ IL1, IL6, IL8, and TNF‐α↓ → Necrosis level of prostatic carcinoma Tc1, Th1, Tc17, and Th17 cells↑ Tumor Tregs↓ CD56+CD16+, CD15+CD16+, F4/80+↑	Jiang et al. (2018)
	In‐vitro	Smoking induced A549 cells (human alveolar basal epithelial cells)	1, 10, 100 nM, and 1, 10 μM	24 h	Protected from oxidative stress‐induced lung cancer and improved genome stability	OGG1↑ NEIL1, NEIL2, NEIL3↑ Cx43↑ SR‐B1 mRNA↑	Cheng et al. (2020)
		Human pancreatic cancer cells (PANC‐1)	0.25 or 0.5 μM	24 h	Induced apoptosis in PANC‐1 cells and prevented pancreatic cancer	Intracellular and mitochondrial ROS↓ NF‐κB↓ IκBα phosphorylation↓ Cleaved caspase‐3, Bax↑ Bcl‐2↓, MMP↓ cIAP1, cIAP2, and survivin↓	Jeong et al. (2019)
		Human prostate cells (PCa cells)	0.5, 1, 2.5, 5, 10 and 20 μM	96 h	Regulated proliferation and apoptosis, and prevented prostate cancer	Cell proliferation↓ Cell cycle arrest↑ PPARγ, RXR, Tp53↑ Bax↑, Bcl‐2↓	Soares et al. (2014)
		Human primary prostatic epithelial (PrE) cells	2 μmol/L	48 h	Prevention initiation, promotion, and/or prostate cancer progression	GSTP1, GSTO1, and SQR↑ Cell proliferation↓ AKT/mTOR pathway↓ TNF‐α signaling↓ MAPK pathway↓ Androgen signaling↓ Apoptosis↓	Qiu et al. (2013)
Cardioprotection	Animal studies	30 adult male rats (*Rattus norvegicus*)	1 mg/kg	4 weeks	Ameliorated cardiac disorders	Lipid fractions, LDL‐C↓ HDL‐C↑ Hyperactivity of LDH, CK, AST, ALT↓	Hassan and Edrees (2004)
		Adult male albino Wistar rats	1 mg/kg	31 days	Reduced myocardial ischemia–reperfusion injury	Arterial pressure and heart rate↑ GSH, GSH‐Px↑ CKMB isoenzyme↑ LPO↓	Bansal et al. (2006)
		Male Sprague Dawley rats	40 mg/kg. BW/day	28 days	Improved the cardiac function and ventricular remodeling	P38 activation↓ MMP‐9, type I collagen↓ Collagen volume fraction in peri‐infarcted zone↓ Cardiac and ventricular function↑	Wang et al. (2014)
		New Zealand male rabbits	42.6, 85.2, and 127.8 ppm	12 weeks	Lowered blood cholesterol levels and protected from CVD	Serum TC, LDL, TG, ApoB↓ Serum HDL and ApoA1↑ Atherosclerotic plaques formation↓ Hepatic HMG‐CoA reductase↓ Cholesterol excretion↑	Verghese et al. (2008)
		Forty male New Zealand white rabbits	4 and 12 mg/kg. BW/day	8 weeks	Protected from atherosclerosis	Serum TC, TG, LDL‐C, oxidized LDL↓ IL‐1↓ MDA↓, TAC, NO↑ Atherosclerotic plaques formation↓	Hu et al. (2008)
		New Zealand white rabbits	5 mg/kg. BW/day	4 weeks	Reduced blood cholesterol levels and prevented the risk of cardiovascular diseases	Plasma lycopene↑ Serum lipid, TG, LDL‐C↓ LDL/HDL ratio↑ Aortic cholesteryl ester↓	Lorenz et al. (2012)
		Isoproterenol treated‐male adult albino Wistar rats	10 mg/kg. BW/day	30 days	Attenuated isoproterenol‐induced apoptosis and myocardial infarction	SBP, DBP, AP↓ C‐reactive protein, myeloperoxidase↓ Nitrite↓, Infarction area↓ Caspase‐3 protease↓ DNA fragmentation↓ Electrolyte imbalance↓	Upaganlawar et al. (2012)
	In vitro	Human platelets	2 to 12 μmol/L	3 min	Prevented platelet aggregation and thrombosis	cGMP and nitrate formation↑ Phospholipase C activation, phosphoinositide breakdown, and thromboxane B2 formation↓ Latency period for platelet‐plug formation↑ Platelet aggregation inhibition↑	Hsiao et al. (2005)
		Normolipidemic‐overnight fasting volunteers' blood	0–200 μmol/L	3 h	Protected LDL from oxidative reactions and ameliorated atherosclerosis	Copper catalyst‐induced LDL‐Ox↓ TBARS and lipid peroxidase formation↓	Safari (2007)
Neuroprotection	Animal studies	Male HDF‐induced Sprague–Dawley rats	4 mg/kg	16 weeks	Prevented learning and memory impairments and attenuated the reduction in dendritic spine density	TG and LDL↓, Escape latencies↓, Dendritic spine density↑	Wang et al. (2016)
		OXL‐induced Sprague Dawley rats	4 mg/kg. BW/day	4 days	Reduced the central and peripheral nerve injuries in OXL‐induced brain and sciatic tissue	ATF6, GRP78, PERK, IRE1↓ NCAM↑ GFAP↓, BDNF↑	Celik et al. (2020)
		Male Wistar rat	4 mg/kg	10 weeks	Prevented oxidative stress, inflammation in the brain, and attenuated learning and memory impairments	Plasma insulin and HOMA‐IR↓ Hippocampal expression of IR, IGF‐1R, PI3K, and p‐AKT protein↓ SOD, CAT, GPx, GSH↑ ROS, LPO, and carbonyl proteins↓ TNF‐α, IL‐1β, and NF‐κB p65↓ PPARγ↑ AchE↓, Ach content↑	Yin et al. (2014)
		Male Wistar rats	2.5 and 5 mg/kg	21 days	Prevented cognitive impairment and protected mitochondria from oxidative damage	Mitochondrial enzymes activities, cell viability↑ SOD, CAT, and GSH↑ LPO, Nitrite↓ AchE activity↓ TNF‐α and IL‐6, caspase‐3↓ BDNF↑	Prakash and Kumar (2014)
		PCBs‐induced male albino Wistar rats	4 mg/kg	30 days	Attenuated nitrosative stress and protected from neuronal damage	→AchE, creatine kinase, nNOS↓, 3‐nitro‐tyrosine↓ Nitrite↓	Janani et al. (2012)
		Male C57BL/6 mice	20 mg/kg	7 days	Protected brain from oxidative and ischemic injury and attenuated apoptosis	Neurological score↑ Neuronal apoptosis, oxidative stress↓ ROS↓, GSH↑ Nrf2/HO‐1 signaling pathway activation↑	Lei et al. (2016)
		Aluminum‐induced male Wistar rats	4 mg/kg	90 days	Inhibited oxidative stress‐induced inflammation and apoptosis and protected from hippocampal lesions	Hippocampal coefficient↑ Escape latency↓ No. of crossings platform position↑ Normal pyramidal neurons↑ ROS↓, MDA↓, 8‐OHdG↓ GSH, SOD↑, p53, Cyt c, caspase‐3↓ Bax↓, Blc‐2↑ IL‐1β, TNF‐α and IL‐6↓ NF‐κB p65↓, Nrf2↑ HO‐1, NQO1, GCLC and SOD1↑	Cao et al. (2019)
		Female Wistar rats	20 or 40 mg/kg	8 weeks	Reduced oxidative stress and inflammation; and eliminated obesity‐induced brain dysfunction	Lipid accumulation in cerebrum↓ AchE, ADA, MAO‐A, 5′‐nucleotidase, and NTPDase↓ IL‐1β and IL‐6, and NF‐κβ p65↓	Ugbaja, Ugwor, et al. (2021)
		Sprague Dawley rats	5, 10, or 20 mg/kg	7 days	Protected neurons from hypoxic–ischemic (HI) brain injury and associated inflammation and apoptosis in brain	TNF‐α, IL‐18, IL‐6, and iNOS mRNA↓ Bax, cleaved Caspase‐3↓, Bcl‐2↑, P65↓ HO1, Nrf2↑, Nrf2/NF‐κB pathway↑ Extent of neuron degeneration and necrosis↓ Nissl bodies and neurons↑ Mean escape latency↓ Crossing frequency↑	Fu et al. (2020)
		Male C57BL6/J mice	10 and 20 mg/kg	12 days	Attenuated the SPS‐induced anxiety‐like behaviors and PTSD‐like behavioral deficits	BDNF expression↑ Time and entries in open arms in the EPM↑ TNF‐α, IL6, and IL‐1β↓ MDA and nitrite↓, GSH↑	Li et al. (2020)
		Male Sprague–Dawley rats	40 mg/kg	40 days	Attenuated neurological deficits, brain water content, BBB disruption, neuronal apoptosis, and neuroinflammation	Neurological score↑ Brain water content, blood–brain barrier permeability, brain edema↓ Cleaved Caspase 3**↓** TNF‐α, IL‐1β, and ICAM‐1↓	Wu et al. (2015)
		Male Sprague–Dawley rats	5, 25, 45, 65, and 85 mg/kg. BW/day	4 weeks	Exhibited anti‐injury properties against hyperlipidemia and antiapoptotic properties in the brain	Serum TC, TG, LDL‐C↓ IL‐1, TNF‐α↓, ox‐LDL↓ Content of Glu, DA↓ Caspase‐3↓ LDLR, NGF, GABA, 5‐HT, GABA_A_, and 5‐HT_1_ levels↑ Bcl‐2 and hippocampal neuron quantity (CA1 and CA3 areas)↑	Yang et al. (2018)
		Male Sprague–Dawley rats	6 mg	14 days	Nano‐liposome encapsulation increased the efficiency of lycopene and protected the brain from I/R injury	Cerebral infarction↓ NOS and NOX2↓ Bcl‐2↑, Caspase‐3↓ MAPK‐JNK, IL‐6↓, FPN1↑	Zhao et al. (2018)
		Female Wistar rats	0.24 and 0.48 mg/kg	2 weeks	Prevented alteration in neuroenzymes functions, oxidative damage, and neuroinflammation	AchE, ADA, MAO‐A, NTPDase↓ MDA↓, GSH↑ TNF‐α, IL‐6 and IL‐1β↓, IL‐10↑ TLR4/NF‐κB‐p65↓	Ugbaja, James, et al. (2021)
		Male Sprague Dawley rats	5, 10, or 20 mg/kg	7 days	Attenuated oxidative damage, mitochondrial dysfunction, cell apoptosis, and protected from spinal cord injury	MDA↓, SOD, GSH‐Px↑ Cyt b, Tfam, ΔΨm↑ Caspase‐9, cleaved caspase‐3 and Bax↓ Bcl‐2↑, Cyt C↑	Hu et al. (2017)

In‐vitro	SH‐SY5Y cells	1 to 10 μm	2 h	Protected neuroblastoma cells from oxidative stress and endoplasmic reticulum stress, and prevented apoptosis	Bcl‐2↑, Bax, and cleaved Caspase 3↓ MDA, 8‐OHdG, and protein carbonyls↓ CHOP and PERK‐eIF2α cascade↓ ER stress↓	Ou et al. (2020)

SH‐SY5Y cells	2.0 or 4.0 *μ*moL/L	2 h	Protected neuroblastoma cells from apoptosis, oxidative stress, and mitochondrial dysfunction	SOD, CAT↑ Caspase‑3 activation↓ AIF translocation↓ Release of Cyt c and AIF↓ MPTP opening, Bax↓ Bcl‑2, MMP↑	Feng et al. (2016)
Renoprotection	Animal studies	OTA induced‐male Sprague–Dawley rats	5 mg/kg/day	14 days	Protected from nephrotoxicity and oxidative stress	BUN, SCr, and plasma electrolytes↓ GPx1 and GSH↑	Palabiyik et al. (2013)
		Male Wistar rats	Lyc‐O‐Mato 6%	6 weeks	Protected from oxidative stress and inflammation in the kidney	Insulin resistance↑ RAGE, and TNF‐α↓	Pierine et al. (2014)
		Male Wistar rats	6 mg/kg. BW/day	10 days	Protected from nephrotoxicity and lipid peroxidation	SCr, BUN↓ MDA, 8‐isoprostane↓ Bax↓, Bcl2↑ Renal HSP60 and HSP70↓	Dogukan et al. (2011)
		Male albino rats	1 mg/kg. BW/day	30 days	Protected kidney from pesticide toxicity	Weight gain, food intake, and absolute kidney weight↑ Serum TNF‐α↓ Congestion, hemorrhages↓ Tubular necrosis, degeneration, dilation, vacuolization↓ Hypercellular and swollen glomerular structures↓ Vessel wall thickness and interstitial fibrosis↓ Collagen deposition↓	El‐Gerbed (2014)
		Male Wistar‐Albino rats	5 mg/kg. BW/d	15 days	Protected from nephrotoxicity and oxidative damage	BUN, SCr, chloride↓ MDA↓, GSH↑ GST, CAT, GSH‐Px, SOD, and G6PD↑ Sodium, phosphorus↑	Yilmaz et al. (2018)
		Colistin‐induced female Kunming mice	5 or 20 mg/kg. BW/d	7 days	Protected from nephrotoxicity, oxidative damage in kidney	BUN, SCr↓ Tubular apoptosis/necrosis↓ Lipid peroxidation↓ Nrf2, HO‐1↑, GSH, CAT, SOD↑ NF‐κB mRNA↓	Dai et al. (2015)
		Renal IR injured‐Swiss Albino adult male mice	10 mg/kg (IP)	30 min	Protected from ischemic‐reperfusion injury and attenuated acute kidney injury	BUN, SCr, plasma NGAL↓ Notch2/Hes 1, TLR 2, IL‐6, Bax, F2‐isoprostane↓	Hussien et al. (2020)
		AFB1‐induced male Kunming mice	5 mg/kg	30 days	Protected kidney from oxidative damage, and enhanced the antioxidant capacity in kidney	BUN, SCr↓ MDA, H_2_O_2_↓ SOD and CAT↑ NQO1, SOD1, GSS, GCLM, and GCLC↑	Yu et al. (2018)
		Wistar rats	6 mg/kg	12 days	Protected from nephrotoxicity and acute kidney injury	BUN, SCr↓ MRP2, and MRP4↓ OAT1, OAT3, OCT1, and OCT2↑	Erman et al. (2014)
		STZ‐induced male Sprague–Dawley rats	20 mg/kg. BW/day	8 weeks	Prevented diabetic nephropathy and improved antioxidant capacity of the kidney	BUN, urea protein, and Cr↓ TC, TG, LDL↓, HDL↑ MDA, CTGF↓, SOD↑ Akt/PKB phosphorylation↑	Li et al. (2014)
		Adult (non‐breeding) male Wistar rat	4 mg/kg. BW/day	8 weeks	Protected from nephrotoxicity and renal damage	BUN, SCr↓ MDA↓, GSH, GSH‐Px, and SOD↑ Mean area percentage, PAS +ve material optical density↑, Desmin‐positive cells (%)↓ Bcl2↑	Shalaby and El Shaer (2019)
		Wistar‐Albino female rats	100 mg/kg	45 min	Protected from renal ischemia/reperfusion injury	BUN, SCr↓ MDA↓, GSH↑ Brush border loss, tubular vacuolization, necrosis, dilatation↓	Kaya et al. (2015)
		Male Kunming mice	5 mg/kg	21 days	Attenuated autophagy and oxidative stress in the kidney and protected from nephrotoxicity	Tubular epithelial cell swelling↓ MDA, H_2_O_2_↓, SOD, GPx, CAT↑ Nrf2 activation↑ LC3II/LC3I, ATGs, Belin1 and p62↓ p62/SQSTM↓ AV formation, LC3 aggregation↓ pAMPK/AMPK↓ NQO1, HO1↓	Lin et al. (2018)
		Male Sprague–Dawley rats	10 mg/kg	21 days	Protected from nephrotoxicity and oxidative damage	SCr, BUN↓ GSH GSH‐Px, CAT↑, TBARs↓ Tubular necrosis, degeneration, dilation, vacuolization↓ Thickened basement membrane↓ Luminal cast formation↓ Inter‐tubular fibrosis↓	Ateşşahin et al. (2007)

*Note*: ↑ = increase; ↓ = decrease/inhibit; → = normal/no change.

Abbreviations: AchE, acetylcholinesterase; ApoA1, apolipoprotein A1; ApoB, apolipoprotein B; Bax, Bcl2 associated X; Bcl‐2, B‐cell lymphoma 2; BDNF, Brain‐derived neurotrophic factor; cIAP, cellular inhibitor of apoptosis protein; CVD, cardiovascular diseases; FPN1, ferroportin‐1; G6PD, glucose‐6‐phosphate dehydrogenase; GFAP, glial fibrillary acidic protein; GLUT, glucose transporter; GPx1, glutathione peroxidase 1; GST, glutathione S‐transferase; IRE1, inositol‐requiring enzyme 1; IκBα, NF‐κ light polypeptide gene enhancer in B‐cells inhibitor‐alpha; MMP, matrix metalloproteinase; MPE, maximum possible effect; NCAM, neural cell adhesion molecule; NEFA, non‐esterified fatty acid; NEIL, Nei like DNA glycosylase; NQO1, NAD(P)H dehydrogenase [quinone] 1; OGG1, 8‐oxoguanine glycosylase; PPARγ, peroxisome proliferator‐activated receptor γ; SIRT, sirtuin; SQSTM1, sequestosome 1; SR‐B1, scavenger receptor class B type 1; TGF‐β1, transforming growth factor beta 1; ZO‐1, zonula occludens‐1; α‐SMA, α‐smooth muscle actin.

**TABLE 4:** Clinical evidence of protective actions of lycopene against age‐related disorders.

Properties	Study subjects	Dose	Duration	Major outcomes	Molecular markers	References
Diabetes prevention	35 patients with T2DM of both sexes aged 54 ± 9 years	10 mg/day	2 months	Prevented long‐term diabetic‐induced complexities, including cardiovascular disease	Serum lycopene↑ Serum MDA↓ MDA‐LDL formation↓ Serum TAC↑ Serum IgM levels↑ Serum anti‐oxidized LDL‐IgG levels↓	Neyestani et al. (2007b)
	32 type 2 diabetes patients, aged 40–60 years	200 g raw tomato/day	8 weeks	Reduced the risk of cardiovascular disease in T2D patients	Systolic and diastolic blood pressure↓ ApoA1↑ ApoB↓	Shidfar et al. (2011)
Cancer prevention	32 patients (mean age = 66.2 ± 6.5) diagnosed with HGPIN	20–25 mg/day	6 months	Reduced HGPIN progression rate to prostate cancer	PSA↓ Plasmatic and prostatic lycopene concentrations↑	Mariani et al. (2014)
	15 men with newly diagnosed prostate cancer	30 mg	3 weeks	Prevented hallmarks of cell proliferation, apoptosis, and prostate cancer	Plasma PSA↓ Surgical margins/extra‐prostatic tissues with cancer, tumors, multifocal and/or diffuse involvement by HGPIN↓ Cx43 level↑ Bcl‐2↑, Bax↓ Plasma IGF‐l and IGFBP‐3↓	Kucuk et al. (2002)
	71 patients with prostate cancer and rising PSA (mean age was 75 years, and mean PSA was 6.5 ng/mL)	15 mg twice daily	6 months	Protected prostate cancer patients with PSA relapse disease and delayed PSA progression rate in prostate cancer	Rate of PSA level rise↓	Vaishampayan et al. (2007)
Skin protection	Twenty Healthy women (phototype I/II, age range 21–47 years)	16 mg/day	12 weeks	Protected from UVR‐induced oxidative stress or photo‐damage (erythema, matrix changes, and mitochondrial DNA damage)	MMP‐1, Fibrillin‐1↓ PCI deposition↑ mtDNA 3895‐bp deletion↓	Rizwan et al. (2011)
	65 healthy volunteers (52 men and 13 women, age range 21–60 years)	Two capsules (5 mg) twice a day	12 weeks	Protected from UV radiation (UVA1 and UVA/B)‐induced skin damage	HO‐1, ICAM‐1, and MMP‐1 mRNA↓	Grether‐Beck et al. (2017)
	30 volunteers (15 male and 15 female, average age 55 years, BMI range 30–35 kg/m^2^)	7 or 30 mg/day	1 month	Improved skin parameters and reduced oxidative stress	GAL‐PUFA formulation↓ Gram‐negative bacteria on skin surface↓ Sebum droplet size↑ Corneocyte damage, corneocyte exfoliation rate↓ IOD and LDL‐Px↓	Wiese et al. (2019)
	10 healthy individuals (5 men and 5 women, age range 21–47 years)	7 mg/day	4 weeks	Prevented skin inflammation, oxidative damage, and acne development	Lycopene bioavailability↑ IOD and LDL‐Px↓	Chernyshova et al. (2019)
	20 women (non‐smokers phototype II or III, age range 20–40 years)	16 mg/day	10 weeks	Protected from UVB‐induced skin damage	Erythema↓	Sokoloski et al. (2015)
Cardioprotection	23 non‐smoking, healthy men (BMI range 19.6–28.1 kg/m^2^, age range 27–40 years)	40 mg/day	14 days	Prevented the risk of atherosclerosis and cardiovascular disease	LDL‐Ox↓ Plasma TBARS↓	Bub et al. (2000)
	12 healthy female volunteers	8 mg/day	3 weeks	Prevented the risk of atherosclerosis, and CVD	8 iso‐PGF2α excretion↓ LDL‐Px↓	Visioli et al. (2003)
	22 healthy, non‐smoking men	37 mg/day	2 weeks	Reduced the risk of CVD	LPO↓ Plasma MDA↓ →PON1	Bub et al. (2005)
	40 patients with grade‐1 HT (age range 30–70 years)	15 mg/d	4 weeks	Reduced the risk of hypertension and CVD	SBP, DBP↓ TBARS↓	Engelhard et al. (2006)
	21 healthy, non‐smoking individuals (5 men and 16 women) (age range 20–49 years, average BMI 23.5 ± 2.3 kg/m^2^)	27 mg/d	3 weeks	Reduced the risk of CVD	TC and T‐LDL↓	Silaste et al. (2007)
	29 healthy individuals (15 females and 14 males) (Average age 27 ± 8 years, BMI range 19 and 24 kg/m^2^)	27,038.2 mcg/meal	2 days	Attenuated lipemia‐induced post‐prandial oxidative, inflammatory responses and reduced the risk of CVD	Plasma glucose, insulin, and lipid concentrations↑ TG↑, LDL‐Ox↓ IL‐6↓	Burton‐Freeman et al. (2012)
	432 individuals (HDL‐C: men <40 mg/dL and women <50 mg/dL; triglyceride concentration: <150 mg/dL)	2 uncooked Roma tomatoes/d	4 weeks	Reduced the risk of CVD	Serum HDL‐C↑	Cuevas‐Ramos et al. (2013)
Renoprotection	120 patients (age range from 20 to 80 years)	25 mg (pre‐treatment)	72 h	Protected from nephrotoxicity in patients with cancer	GFR↑, BUN↓	Mahmoodnia et al. (2017)

*Note*: ↑ = increase; ↓ = decrease/inhibit; → = normal/no change.

Abbreviations: HGPIN, high‐grade prostatic intraepithelial neoplasia; IGF‐1, insulin‐like growth factor 1; IGFBP, IGF binding protein‐3; PON1, paraoxonase 1.

### 4.1 Potentials of lycopene against aging biomarkers

#### 4.1.1 Oxidative stress

Oxidative stress is one of the major biomarkers of the aging mechanism, which occurs when free radical levels cross over the antioxidant levels in the body. Oxidative stress is due to excessive ROS generation, resulting from many cellular and biochemical signaling impairments, which lead to tissue damage, DNA damage, inflammatory pathways activation, apoptosis, and so on (Vatner et al., 2020). This oxidative stress is directly linked to many aging‐related chronic disorders, including cancers, diabetes, renal disorders, cardiovascular disease, and neurodegenerative diseases (Hajhashemi et al., 2010; Vatner et al., 2020). Antioxidant components such as vitamin E, lycopene, and tocopherols can significantly protect against oxidative damage. For example, the antioxidant properties of lycopene have been shown to help keep free radical levels in control and shield the body from several harmful conditions (Fiedor & Burda, 2014).

Many cell studies and pre‐clinical and clinical studies demonstrated that lycopene could protect from oxidative damage through several antioxidative mechanisms, as shown in Table 2 and Figure 2. Studies on human monocytic cell lines (THP‐1) showed that lycopene could inhibit oxidative stress by downregulating ROS production, 8‐OHdG formation, and the expressions of NOX4, NADPH oxidase expressions, Hsp70 and Hsp90 expressions (Palozza et al., 2010; Simone et al., 2011). Another study on lipopolysaccharide (LPS)‐mediated (RAW 264.7) macrophages showed that lycopene could prevent oxidative stress by inhibiting mRNA expressions of iNOS and NO production (Feng et al., 2010). Furthermore, a study on albino rats showed that lycopene could reduce MDA levels, lipid peroxidation, total nitrate/nitrite ratio, and increased total antioxidant capacity (TAC) and antioxidant enzymes SOD and GSH levels (Mansour & Tawfik, 2012). A similar result was also found in a study of HgCL_2_‐induced Wister rats (Yang et al., 2011) and cisplatin‐induced Wistar rats (Sahin et al., 2010), as summarized in Table 2. Sahin et al. (2010) also demonstrated that lycopene could improve antioxidant levels by inducing Nrf2 accumulation and HO‐1 expressions. Likewise, Dai et al. (2015) observed that a 7‐day oral treatment of lycopene restored antioxidation levels in colistin‐induced Kunming mice by increasing GSH, CAT, SOD levels, mRNA expressions of Nrf2, and HO‐1 and lowering NF‐κB mRNA expressions and lipid peroxidation (LPO). Furthermore, clinical studies on both healthy volunteers (Visioli et al., 2003; Zhao et al., 2006) and chronic diseases (T2D and prostate cancer) patients (Chen et al., 2001; Upritchard et al., 2000) reported that lycopene prevented lipid oxidation as well as downregulating oxidative DNA damage (Table 2). In addition, a study proved that the lycopene mycelium powder protected *Drosophila melanogaster* from oxidative stress, delayed longevity, increased reproductivity, and sexual capacity by increasing SOD and decreasing MDA levels (Hu et al., 2013). The considerable evidence suggests that the supplementation of lycopene could be a potential remedy for the participation of oxidative biomarkers in aging and aging‐related chronic disease development.

#### 4.1.2 Inflammation

Inflammation is another critical biomarker of aging, which is a vital part of the immune defense system in any living organism. Acute inflammation protects against pathogenic infection, while chronic inflammation contributes to chronic disorders. Chronic inflammation exacerbates several aging‐related chronic complexities, including metabolic disorders, atherosclerosis, neurological disorders, and cancer (Chung et al., 2019). Several factors, including endoplasmic reticulum stress, inflammasome, HMGB1, the receptor for AGE (RAGE), and toll‐like receptors (TLRs), are associated with the induction of chronic inflammation by triggering the release of several pro‐inflammatory mediators, including IL‐1β, IL‐6, TNF‐α, IFN‐α, and the activation of various pro‐inflammatory cytokines regulatory pathways, including NF‐κB, MAPK, JAK/STAT signaling pathways (Chung et al., 2019; Ferrucci & Fabbri, 2018).

Lycopene exerts excellent anti‐inflammatory properties by inhibiting pro‐inflammatory cytokine release and regulating the inflammatory pathways and co‐factors, evident in many in‐vitro, animal, and human studies, as shown in Table 2 and Figure 3. For example, in vitro studies on LPS‐mediated (RAW 264.7) macrophages showed that lycopene attenuated inflammation by preventing IL‐6 and IL‐1β mRNA expressions, NF‐κB pathway, and inhibiting p38, IκB, ERK1/2, and JNK phosphorylation (Feng et al., 2010; Marcotorchino et al., 2012). Similarly, studies on THP‐1 cell lines demonstrated that pre‐, or post‐treatment with lycopene inhibited inflammation by preventing NF‐κB DNA binding, downregulating NF‐κB/p65 nuclear translocation, and phosphorylation of IκBα, IKKα, ERK1/2, JNK, and p38 MAPKs (Palozza et al., 2010; Simone et al., 2011). Again, another study on LPS‐induced human umbilical vein endothelial cells (HUVECs) demonstrated that lycopene significantly prevented the release of HMGB1 and the expressions of HMGB1‐mediated TNF‐α, sPLA2‐IIA and lowered the expressions of VCAM‐1, ICAM‐1, E‐selectin, TLR‐2, TLR‐4, HMGB1 receptors, and RAGE receptors (Lee et al., 2012).

An animal study on streptozotocin (STZ)‐induced diabetic Wistar rats found that lycopene administration for 10 weeks downregulated STZ‐induced TNF‐α production (Kuhad, Sethi, & Chopra, 2008). Another study on hyperhomocysteinemic Sprague–Dawley (SD) rats fed with lycopene inhibited the expressions of VCAM‐1, MCP‐1, IL‐8, attenuated endothelial dysfunction, and protected from atherogenesis (Liu et al., 2007). Interestingly, studies on alcohol‐fed Fischer rats cautioned about higher lycopene intake that a high dose of lycopene (1.1 or 3.3 mg/kg. BW/day) could increase pro‐inflammatory TNF‐α mRNA expressions and inflammatory foci in the liver (Veeramachaneni et al., 2008).

A clinical study on 53 well‐nourished, healthy elderly person consumption with 330 mL/day of tomato or 47.1 mg lycopene for 8 weeks also demonstrated that a higher intake of lycopene increased the TNF‐α, and IL‐4 levels, though it also lowered IL‐2 levels (Watzl et al., 2000). However, another clinical study on 26 healthy young volunteers demonstrated that 5.7 mg lycopene intake for 26 days significantly lowered TNF‐α production (Riso et al., 2006), which could be suggested as the optimum dose for consumption. The in vitro and in vivo studies on lycopene strongly advocated its prospects as an anti‐inflammatory agent, and this compound could be suggested to prevent inflammation‐mediated aging progressions. Nevertheless, it requires more pre‐clinical and clinical studies to elucidate the modulatory effects of lycopene on inflammatory pathways and biomarkers, and this will aid in determining the clinical requirements of lycopene to prevent inflammatory pathologies.

#### 4.1.3 DNA damage

Antioxidants generally scavenge reactive oxygen species (ROS) and protect the cell membranes from oxidation. Furthermore, a protection strategy against ROS may be facilitated by enzyme, antioxidant, vitamin, and carotenoid compounds. However, the volume of ROS and antioxidants synthesis may be imbalanced due to the destruction of antioxidants by chemical compounds, and that imbalance can cause damage to DNA, lipid, and protein. Therefore, the baleful effect of ROS can be minimized by supplementing antioxidant vitamins and carotenoids (Boyacioglu et al., 2016).

Lycopene is a carotenoid compound that can scavenge ROS, especially singlet oxygen, and thus lycopene plays a vital role in oxidative stresses and cancers. During the scavenging of singlet oxygen, energy usually transfers from singlet oxygen to lycopene and converts it to an energy‐rich triplet state. Moreover, quenching of hydroxyl radicals, nitric oxides, and peroxides may lead to oxidative breakage of lycopene. This is how lycopene protects against in vivo oxidative damage of DNA (Stahl & Sies, 2003). Several studies substantiated the DNA damage preventive role of lycopene. For example, studies on ferric nitrilotriacetate (Fe‐NTA)‐induced rats showed that pre‐treatment by lycopene decreases 8‐oxodGuo level in rats, demonstrating that lycopene molecule vigorously protects against Fe‐NTA‐inducing DNA base oxidation (Matos et al., 2001). Likewise, lycopene administration protected indomethacin‐induced DNA damage in rats (Boyacioglu et al., 2016) and ochratoxin‐A‐inducing DNA damage in hepatic and kidney tissues (Aydin et al., 2013). Other studies also include protection against gamma‐radiation‐inducing DNA damage in hepatocytes (Srinivasan et al., 2007), sodium fluoride‐inducing DNA damage in NRK‐52E kidney cells (Çetin et al., 2021), and deltamethrin‐inducing DNA damage in thyroid cells (Abdul‐Hamid & Salah, 2013). From the literature, it is clear that even though lycopene has higher potency in DNA protection, there is a lack of sufficient evidence of the DNA‐damage preventive mechanisms of lycopene, particularly in humans. Therefore, more intensive studies are required to elucidate the mechanisms, pathways, and consequences associated with the DNA‐damage protective actions of lycopene.

#### 4.1.4 Telomere length shortening

Telomeres are 6‐bp (base pair) repeated sequences of TTAGG located at the end of a chromosome in mammals, which are attached with the help of an enzyme named telomerase (Muraki et al., 2012). The proteins are generally not encoded by telomeres, but they help to protect against chromosome damage when cell divisions occur (Blackburn, 1991). Moreover, telomeres are essential for regulating cell lifespan, and the shortening of telomeres implicates cellular senescence and apoptosis (Blackburn, 2005). Though telomeres length is mainly inherited and heterogeneous (Min & Min, 2017), it is known as one of the biomarkers of aging (Jiang et al., 2008). Besides, studies have substantiated that shorter telomere length is related to a higher risk of aging and age‐related disorders (Aubert & Lansdorp, 2008). However, an increase in oxidative stress has a crucial role in telomeres length shortening associated with the conditions mentioned above (Babizhayev et al., 2011).

A recent study indicated that serum lipophilic antioxidants positively affect leukocyte telomeres length. In this study, *trans*‐lycopene, along with α‐carotene, β‐carotene (*trans*+*cis*), β‐cryptoxanthin, and combined lutein/zeaxanthin were analyzed to determine these antioxidants association with telomere length. As a result, the telomere length increased when these antioxidant levels in the serum increased (Mazidi et al., 2018). Besides, another study suggested that, statistically, non‐provitamin‐A carotenoid compounds, such as trans‐lycopene and combined lutein/zeaxanthin, do not significantly affect telomeres length (Min & Min, 2017). Therefore, further investigations are required to eliminate the controversies and establish a claim on behalf of lycopene.

#### 4.1.5 Cellular senescence

Cellular senescence is a process that can impose proliferation arrests on a cell as a response to numerous stressor agents. It is an important biomarker in aging and aging‐related disorders and has become a vital target for therapeutic utilization (Childs et al., 2015). Unfortunately, the studies of lycopene on cellular senescence are minimal.

Recently, a study was conducted to demonstrate the effect of lycopene on p38 MAPKs activity of endothelial progenitor cells (EPCs), which were cultured with high glucose (HG), and the result of this study demonstrated that lycopene could prevent HG‐inducing EPC injuries due to the inhibition of p38 MAPK activity (Zeng et al., 2014). On the other hand, previous studies showed that high glucose could accelerate the onset of EPCs senescence by activating p38 MAPK (Piconi et al., 2006). Hence, it can be said that lycopene's capability to block the activation of p38 MAPK may lead to the inhibition of EPCs senescence (Zeng et al., 2014). However, the lack of knowledge on lycopene in cellular senescence confers a poor understanding of the prospects of lycopene administration against this aging biomarker. Therefore, further studies are required to substantiate the role of lycopene in cellular senescence and its impacts on the aging process.

#### 4.1.6 DNA methylation

DNA methylation is an essential epigenetic gene alteration involving numerous cellular regulation processes. These processes are embryogenesis, chromatin structures, transcription, lyonization, genomic imprinting, and chromosome stability. Moreover, several human diseases are associated with abnormal DNA methylation and the aforementioned roles (Robertson, 2005). Therefore, many extensive studies have been conducted to understand the role of lycopene in DNA methylation. An initial study indicated that lycopene does not alter the DNA methylation of glutathione S‐transferase P1 (GSTP1) promoter in LNCaP cell lines of prostate carcinoma (Liu & Erdman, 2011). However, lycopene also induced GSTP1 expressions and downregulated androgen signaling in human primary prostatic epithelial (PrE) cells (Qiu et al., 2013). Later, it was manifested that lycopene supplementation can significantly decrease the methylation level of GSTP1 in androgen‐independent PC3 cell lines of prostate carcinoma, whereas demethylation of GSTP1 or upregulated GSTP1expressions was not seen when lycopene was supplemented in androgen‐dependent LNCaP cell lines. Therefore, the results from these studies demonstrated that lycopene's protection effect on prostate carcinoma could vary between androgen‐dependent and androgen‐independent cell lines (Fu et al., 2014). Apart from these, lycopene has been proven to cause partial demethylation and restoration of GSTP1 expressions in different breast carcinoma cell lines (King‐Batoon et al., 2008). Therefore, further studies should be conducted to evaluate the role of lycopene in DNA methylation and its potential to prevent aging progressions.

### 4.2 Potentials of lycopene against aging‐related chronic diseases

#### 4.2.1 Obesity

Obesity, sedentary behavior, and a lack of physical activity are all common in adults (Martínez‐González et al., 1999). Obesity and metabolic syndromes are proven to play a role in premature death, especially against type‐2 diabetes and cardiological diseases. Obesity accelerates aging by disrupting metabolic pathways, as demonstrated by the biochemical association between caloric restriction and longevity (Bentley et al., 2018). It alters glucose, amino acid, and fatty acid metabolism, resulting in decreased insulin sensitivity and, as a result, a reduced capacity to respond to energy supply (Johnson et al., 2009). Thus, obesity can directly contribute to the aging processes, including the metabolic effects of aging‐altered mitochondrial signaling and metabolism and the nutrient‐signaling pathway's degraded functions that maintain the balance between insulin and glucagon in blood glucose (Riera & Dillin, 2015).

Several animal models demonstrated that lycopene lowered lipid levels in the blood, hepatic lipid accumulation, prevented weight gain, reduced hepatocytes and adipocyte size, and upregulated PPARγ mRNA expression (Table 3). Lycopene appears to inhibit adipocyte‐macrophage crosstalk, thus preventing obesity‐related adipose inflammation. Furthermore, the findings showed that lycopene reduces obesity‐induced adipose tissue inflammation by controlling macrophage polarization and inhibiting adipocyte hypertrophy, further improving insulin resistance and fatty liver (Chen, Ni, et al., 2019). A 12‐week study on Swiss albino mice showed that lycopene prevented weight gain and adiposity, promoted adipose tissue fat mobilization, and downregulated insulin resistance through downregulating total triglycerides (TG) level in serum, systemic adiposity, improving hepatic glucose/lipid metabolism, and accelerating glucose clearance and insulin sensitivity, respectively (Singh et al., 2016). In addition, another study on C57BL/6J mice with a minimal dose of lycopene prevented obesity by showing a wide range of mechanisms. For example, lycopene inhibited fat accumulation in adipose tissue and improved lipid metabolism by blocking the expressions of lipogenesis genes (*Fas*, *Acaca*, *Pparγ*, *Srebp1c*, and *Pparg*) and upregulating lipidolysis‐related genes expressions, including thermogenic genes (*Pgc1α*, *Prdm16*, *Ucps*, and *Ebf2*) and mitochondrial functional genes (*Cox5b*, *Cox8b*, *CoxII*, *Cycs*, and *Sirt1*). The study also demonstrated that lycopene inhibited autophagy‐mediated lipid accumulation by downregulating autophagy gene expressions (*Atg7*, *Atg14*, *P62*, *Lc3*, and *Beclin*). The study further showed that lycopene improved insulin resistance by lowering the expressions of *Leptin* and increasing the mRNA expressions of *Glut1* and *Glut4*, as well as downregulating intestinal inflammation and intestinal leakage by inhibiting inflammatory biomarkers (IL‐6, IL‐1β, TNF‐α, iNOS, and Cox‐2) and increasing the expressions of *Zo‐1*, *Claudin‐1*, and *Occludin*, respectively (Wang et al., 2019). Again, another study on C57BL/6J mice showed that lycopene improved adipose tissues mobilization, glucose homeostasis, lowered HOMA‐IR index, triglycerides, 8‐iso‐PGF2α, and NEFA concentrations, downregulated the expressions of PPARγ mRNA (*ap2*, *Cd36*, *Lpl*), and lipogenesis genes (*Fasn* and *Acaca*) expressions, and inhibited adipocyte hypertrophy and inflammatory biomarkers (Fenni et al., 2017). A study on Wister rats demonstrated that lycopene could inhibit obesity‐related complications; for example, it could prevent weight gain and liver weight increment. It also improved serum lipid and glucose/insulin profile by lowering serum cholesterol, TG, Apo‐B, LDL‐c, and increasing serum HDL‐c levels, improving lipid metabolism by increasing hepatic PPAR‐γ levels. Lycopene also prevented obesity‐induced oxidative stress, inflammation, and fibrosis in the liver by increasing antioxidant enzymes (SOD, CAT, GSH GPx, and GR) levels, reducing MDA, NO levels, inhibiting inflammatory biomarkers (IL‐1β, TNF‐α, and MPO), and downregulating fibrosis markers (TGF‐β1 and α‐SMA) in the liver. In addition, lycopene prevented obesity‐induced cardiac complications by lowering atherogenic index, serum lactate dehydrogenase (LDH), and creatine kinase levels (Albrahim & Alonazi, 2021). The wide range of actions against obesity biomarkers advocated the potencies of lycopene to ameliorate aging‐related metabolic disorders. Therefore, lycopene could be suggested as a prospective phytomedicine to prevent obesity and obesity‐induced pathobiologies.

#### 4.2.2 Diabetes

Diabetes mellitus is a group of diseases in which the body loses its blood sugar control capacity. Insulin enables glucose transfer from the bloodstream into cells, which are used as fuel. Individuals may suffer from different types of diabetes, such as type‐1 diabetes caused by insufficient insulin production or type‐2 diabetes (inability to use insulin properly), or both (which occur with several forms of diabetes). Since glucose in the blood cannot reach cells efficiently in diabetes, blood glucose levels remain elevated (Siddiqui et al., 2013). Potential explanations for reduced insulin efficacy with aging include increased abdominal fat mass, reduced physical activity, sarcopenia, mitochondrial dysfunction, hormonal changes (i.e., lower insulin‐like growth factor‐1 [IGF‐1] and dehydroepiandrosterone), and increased oxidative stress and inflammation (Goulet et al., 2009).

Several studies substantiated lycopene as an antidiabetic agent by exhibiting multimechanistic modes of action against diabetes. For instance, Imran et al. (2020) discussed the potentials of lycopene and described that lycopene could prevent the risk of diabetes by lowering MDA level, serum nitrate‐nitrite, glycated hemoglobin, and C reactive protein levels; downregulating RAGE receptor, NF‐қB, MMP‐2, and Bax proteins expression; improving Bcl‐xL and Bcl‐2 levels; and increasing and enhancing antioxidant enzymes activities.

Lycopene studies on animal models and clinical studies demonstrated that it could be used to prevent and treat diabetes, as summarized in Tables 3 and 4. A study on SD rats‐derived endothelial progenitor cells showed that lycopene had been proven to rescue the S‐phase of cell cycle arrest and reduce apoptotic rates and autophagy reactions on endothelial progenitor cells of type‐2 diabetes mellitus (T2DM) rats (Zeng et al., 2017). In a study on STZ‐induced diabetic mouse models, lycopene downregulated diabetes‐associated pancreatic injuries, reduced glucose levels in urine and blood, and upregulated serum insulin levels (Ozmen et al., 2016). Studies on albino rats demonstrated that lycopene prevented the risk of type 2 diabetes mellitus and attenuated diabetic neuropathy by preventing oxidative stress in pancreatic tissue by increasing SOD and GSH‐Px, lowering MDA levels, improving glycolipid metabolism by increasing serum HDL, insulin levels, lowering serum glucose, TG, TC, LDL, Gly‐LDL, cholesterol, and GHb level, and inhibiting TNF‐α and NO generation (Kuhad, Sharma, & Chopra, 2008; Yin et al., 2019).

Moreover, a clinical trial of lycopene suggested that consuming lycopene at a 10 mg/day dose for 2 months can reduce the long‐term complication of T2DM by upregulating total antioxidant capacity (TAC) level, restricting MDA and MDA‐modified LDL formation and serum anti‐oxidized LDL IgG levels, and increasing serum IgM1 level (Neyestani et al., 2007a, 2007b). Another clinical study demonstrated that consumption of raw tomato (200 g/day) could prevent type 2 diabetes‐associated cardiovascular diseases by lowering systolic and diastolic blood pressure, upregulating ApoA1, and downregulating ApoB levels (Shidfar et al., 2011).

An epidemiological survey of 24,377 adult people through 24 h dietary recall method and their health examination revealed that non‐diabetic patients consumed more lycopene than diabetic patients (Quansah et al., 2017). A cross‐sectional study on 111 T2DM individuals indicated that a greater risk of diabetic retinopathy correlates with reduced levels of lycopene. This study also suggested that diabetic retinopathy risks can be modulated by adding more lycopene to the diet (Brazionis et al., 2008). Another cross‐sectional study on 1978 pregnant women substantiated the inverse relation between lycopene consumption and the risk of gestational diabetes mellitus (Gao et al., 2019). The evidence of the translational success of lycopene administration against diabetes strongly supported its therapeutic application to diabetic patients to attenuate diabetes and diabetes‐induced pathologies.

#### 4.2.3 Cancer

Lycopene is a potent anti‐cancer agent, which showed excellent anticarcinogenic properties in numerous in vitro and in vivo studies, summarized in Tables 3 and 4 and Figure 4. Laboratory research proved that lycopene could inhibit cancer cell proliferation, including lung cancer, breast cancer, prostate cancer, and endometrial cancer (Levy et al., 1995). An in vitro study demonstrated that lycopene could protect A549 cells (human alveolar basal epithelial cells) from oxidative stress‐induced lung cancer and improve genome stability by inducing 8‐oxoguanine DNA glycosylase (OGG1) expressions and improving Nei‐like DNA glycosylases (NEIL1, NEIL2, NEIL3), gap junction protein (Cx43), and SR‐B1 mRNA expressions (Cheng et al., 2020). Lycopene also prevented pancreatic and prostate cancer by regulating apoptosis by inducing the *Bax* gene and downregulating *Bcl‐2* gene expression, as summarized in Table 3 (Jeong et al., 2019; Soares et al., 2014). Likewise, it prevented breast cancer by lowering cell proliferation‐inducing apoptosis and upregulating the expressions of p53 and Bax mRNAs in MCF‐7 cells (human breast carcinoma cell line) (Peng et al., 2017). Another study demonstrated that HL‐60 cell lines are inhibited with lycopene supplementation (Zhang et al., 2003), and cell cycle continuation is slowed in the G_0_/G_1_ phase, along with apoptotic induction (Amir et al., 1999). Similarly, lycopene inhibited the growth of breast and endometrium cancerous cell lines by arresting the G1 phase of the cell cycle and reducing the activity of protein kinases, mostly the cyclin‐dependent kinases (Nahum et al., 2001). An organotypic cell culture study showed that lycopene suppressed KB‐1 cell growth by causing a dose–response reduction of PCNA. The study also reported that lycopene inhibited the carcinogenic compound 3‐methylcholanthrene‐induced cancerous cell line formation (Livny et al., 2003). Lycopene supplementations considerably abated the STAT3 expression in ovarian cell lines (Cataño et al., 2018) and HGC‐27 cell lines, along with upregulated LC3‐I and phosphorylated‐ERK expressions (Zhou et al., 2016). Moreover, a study on human liver adenocarcinomas metastasis showed that lycopene suppresses the metastasis of the SK‐HEP‐1 cell line by NOX‐4 mRNA expression inhibition and the reactive ROS intracellular activity inhibition (Figure 2) (Jhou et al., 2017). Lycopene is also used to treat colorectal cancer cells in humans, and the introduction of lycopene decreases the prostaglandin E2 and nitric oxide levels (Cha et al., 2017). Moreover, research on prostate cancer indicated that lycopene could upregulate the expressions of the *BCO2* gene while downregulating the growth of the androgen‐sensitive cells (Gong et al., 2016).

Animal models can substantially represent the cancer prevention study; henceforth, lycopene was also studied in various animal models to recapitulate its cancer prevention efficacy. In rat models, lycopene suppressed the proliferation of the C6 glioma cell line when transplanted via retinoid or carotenoid class chemical compounds (Wang et al., 1989). Again, malignant ascites tumor is inhibited in mice when lycopene is injected intraperitoneally in them (Lingen et al., 1959), and lycopene possibly diminishes the carcinogenic property of procarcinogenic compound 7,12‐dimethylbenz(a)anthracene in hamster models (Bhuvaneswari et al., 2005, 2002). Lycopene also shows chemoprotective action in B6C3F1 mice by inhibiting the commencement of lung carcinoma with diethyl nitrosamine, methyl nitrosourea, and symmetrical dimethylhydrazine (Kim et al., 1997). Another rat model study showed that lycopene inhibited methyl nitrosourea‐inducing prostate cancer (Bhuvaneswari & Nagini, 2005).

Moreover, several in vivo studies corroborated that lycopene can be synergistic with oleoresin compounds to inhibit carcinogenic compounds (Narisawa et al., 1996, 1998; Okajima et al., 1998; Watanabe et al., 2001). For example, in a study on the hamster buccal pouch model, lycopene at a 5 mg/kg dose was highly effective in suppressing this carcinogenesis (Chandra Mohan & Nagini, 2003). In addition, a study on Balb/c mice demonstrated that lycopene could prevent inflammatory response‐mediated prostate cancer progression by lowering tumor volume, tumor Tregs, tumor growth, inhibiting proinflammatory cytokines (IL‐1, IL‐6, IL‐8, and TNF‐α) release, increasing inflammatory tumor cells (Tc1, Th1, Tc17, and Th17 cells), expressions of NK immunity cell marker (CD56+CD16+), macrophage immunity cell marker (F4/80+), and neutrophil immunity cell marker (CD15+CD16+) (Jiang et al., 2018). Similar to animal studies, lycopene also exerted its protective role in clinical studies. For instance, a study on 46,719 males with prostate cancer reported that lycopene showed protective mechanisms against prostate cancer by inhibiting the fusion of *TTSPs* and *ERG* genes (Graff et al., 2016). Furthermore, a few clinical trials on prostate cancer patients reported that lycopene could prevent prostate cancer by limiting prostate‐specific antigen (PSA) concentrations and preventing apoptosis in the prostate, as shown in Table 4. The wide range of evidence on behalf of lycopene suggests it could be a potent anticancer agent. Therefore, the therapeutic application of lycopene for treating cancer should be prioritized. Furthermore, additional studies are required to elucidate the toxicity, required dose, and dosage of lycopene prior to recommending lycopene for cancer treatment.

#### 4.2.4 Skin aging

Skin is a membranous layer between the body and the external world. Apart from safeguarding the physique against loss of water and microorganism infection, it plays a significant cosmic role (Blanpain & Fuchs, 2006). As the most voluminous organ exposed to the external environment, the skin is affected by intrinsic and extrinsic aging factors. Aging caused by intrinsic factors is a natural physiologic phenomenon that ends in dry, thin skin with fine wrinkles and progressive dermal degradation. On the contrary, extrinsic aging is a function of outer environmental parameters such as smoking, air pollution, inadequate diet, and sunlight exposure, resulting in elasticity loss, coarse wrinkles, rough‐textured appearance, and laxity (Krutmann et al., 2017; Mora Huertas et al., 2016). These clinical signs ultimately lead to skin aging progression. This aging process affects the phenotype of dermal cells as well as functional and structural changes in extracellular matrix materials such as elastin and collagen (Zhang & Duan, 2018).

As a dynamic process, the aging clock is unstoppable, and numerous morphological and pathophysiological factors influence it. However, increasing lycopene intake can improve collagen health in the skin, preventing fine lines and wrinkles. Due to its reducing properties, lycopene is a highly effective antioxidant that can slow aging by neutralizing ROS, which has already been developed. Since ROS induces the MAPK pathway, it results in the rise of MMP output, which degrades collagen (Zhang & Duan, 2018). Moreover, like most other carotenoids, lycopene effectively quenches singlet oxygen and traps peroxyl radicals (Sies & Stahl, 1995). Peroxyl radicals are endogenous ROS; both have the potential to interact with biologically essential macromolecules, such as protein, lipid, and DNA, and impair their physiological activities (Amir et al., 1999; Nahum et al., 2001), and these interactions are thought to be the precursors to age‐related macular degeneration. However, plasma lycopene levels generally decrease strikingly as we age, and elderly individuals have statistically lower blood lycopene concentrations than younger individuals of similar racial and dietary backgrounds (Semba et al., 2010). Therefore, higher lycopene supplementation is recommended for protection against skin aging.

Though there is limited evidence of the dermal protective actions of lycopene in animal models, a study on Swiss albino mice revealed that lycopene protected skin from photoaging by inducing antioxidant enzymes CAT and GSH and collagen content in skin and lowering TBARS levels (Shah & Mahajan, 2014). In addition, lycopene supplementation also prevented keratinocyte carcinomas in SKH‐1 hairless mice by downregulating tumor numbers in the skin, as summarized in Table 3.

A significant number of clinical trials revealed that lycopene could improve skin parameters and prevent several UV radiation‐induced skin diseases, including erythema, oxidative damage, and inflammation in the skin, summarized in Table 4. For example, a clinical trial on healthy women demonstrated that 16 mg of lycopene administration daily for 12 weeks protected them from photo damages, including erythema, matrix changes, and mitochondrial DNA damage by downregulating MMP‐1, Fibrillin‐1, upregulating PCI deposition, and inhibiting mtDNA 3895‐bp deletion (Rizwan et al., 2011). Similarly, a placebo‐controlled, double‐blinded, randomized, crossover study on 65 healthy volunteers observed that lycopene‐rich tomato nutrient complex (TNC) could protect from UVA1 and UVA/B radiation‐induced skin damage by inhibiting the expressions of HO‐1, ICAM‐1, and MMP‐1 mRNA (Grether‐Beck et al., 2017). The outcomes in clinical and pre‐clinical studies strongly represent the prospects of lycopene in skincare. Nonetheless, more in‐depth studies should be done to elucidate the preventive actions of lycopene against various aging‐related skin disorders, including pruritus, eczematous dermatitis, purpura, skin cancer, and many others.

#### 4.2.5 Cardiovascular disorders

Cardiovascular or cardiological diseases are prevalent causes of human health declination and co‐morbidity (Virani et al., 2021). Factors affecting cardiovascular diseases include biological factors, genetic factors, dietary components, oxidative enzymes, antioxidant enzymes, and lifestyles (Verghese et al., 2009). Mostly referred cardiovascular diseases are myocardial infarction or heart attack, heart failure, arterial hypertension, angina, cardiac arrest, stroke, heart failure, coronary artery disease, peripheral artery disease, valvular heart disease, and congenital heart defect (Hasan & Sultana, 2017). Lycopene‐rich foods can be highly beneficial in preventing cardiovascular diseases as lycopene is a potential source of antioxidants (Ruxton et al., 2006). Several in vitro and in vivo studies substantiated the efficacy of lycopene in cardiovascular disease prevention. Furthermore, dietary carotenoids, which contain a significant amount of lycopene, can thwart cardiac and vessel infection (Mozaffarian et al., 2011).

A recent meta‐analysis of the observational study evaluated the efficacy of cardiovascular disease prevention with the dietary intake of lycopene. The meta‐analysis study reported that lycopene could reduce 17% of cardiovascular disease risks when the highest dietary intake is compared with the lowest intake level (Song et al., 2017). A recent study was conducted to comprehend the relationships between lycopene and cardiovascular function, revealing that lycopene supplements or lycopene‐containing foods can reduce the LDL cholesterols level in humans (Costa‐Rodrigues et al., 2018). Furthermore, lycopene‐containing foods also elevate the function of endothelial cells. In addition, reduction of systolic and diastolic blood pressure, downsizing of inflammatory mechanisms, cell adhesion molecules, triacylglycerols lessening, and HDL cholesterols escalation are all proven to be possible connections with lycopene supplements or lycopene‐containing food consumption (Cheng et al., 2017). An in vitro study reported that lycopene could prevent platelet aggregation and thrombosis by upregulating cyclic GMP and nitrate formation, the latency period for the induction of platelet‐plug formation, inhibiting the activation of phospholipase C, phosphoinositide breakdown and thromboxane B2 formation, and accelerating platelet aggregation inhibition (Hsiao et al., 2005). Another study on blood collected from normolipidemic overnight fasting volunteers showed that lycopene prevented oxidation of LDL and recovered from atherosclerosis by inhibiting metal catalyst‐induced LDL oxidative reactions, TBARS levels, and lipid peroxidase formation (Safari, 2007).

Some in vivo and clinical studies also prove the efficacy of lycopene in cardiovascular disease prevention, as shown in Tables 3 and 4. For example, an animal study reported that lycopene attenuated oxidized frying oil‐mediated cardiac disorders by reducing the levels of lipid fractions, LDL‐C, increasing HDL‐C levels, and restricting the hyperactivity of heart enzymes, alanine aminotransferase (ALT), aspartate aminotransferase (AST), creatine kinase (CK), and lactic dehydrogenase (LDH) (Hassan & Edrees, 2004). In addition, a few studies on New Zealand rabbits demonstrated that lycopene significantly prevented the risk factors for cardiovascular diseases, including atherosclerosis, by regulating serum lipid profile, improving LDL/HDL ratio, total antioxidant capacity (TAC), and ApoB levels, inhibiting atherosclerotic plaque formation, hepatic HMG‐CoA reductase activities, and reducing ApoA1, IL‐1 and MDA levels (Table 3) (Hu et al., 2008; Lorenz et al., 2012; Verghese et al., 2008). Moreover, many clinical trials also advocated on behalf of lycopene that an amount of lycopene intake prevented the risk of cardiovascular diseases and associated complications through reducing systolic and diastolic blood pressure, LDL‐oxidation, LDL‐peroxidation, plasma TBARS levels, 8 iso‐PGF2α excretions, and increasing serum HDL‐c levels, demonstrated in Table 4. However, despite the growing supportive data about the benefits of lycopene, controversy at some viewpoints has not been wholly diminished yet. As a result, most studies prioritize the application of tomato‐based food intake instead of lycopene supplementation to manage cardiovascular diseases (Burton‐Freeman & Sesso, 2014). Hence, considering the conflict of interest, existing knowledge gap, and evidence on behalf of lycopene's performance against cardiopathologies, future investigations should focus on the bioavailability of lycopene and its interactions with metabolic pathways to elucidate the pharmacokinetics and prospects of lycopene for clinical administration in the case of patients with cardiovascular diseases.

#### 4.2.6 Neurological disorders

Aging processes significantly contribute to neurological disorders like Alzheimer's and Parkinson's. Several hallmarks of aging play a vital role in the neurodegeneration process, such as genome instability, DNA damage, telomere shortening, epigenetic alteration, proteostasis loss, mitochondrial disease, cellular senescence, dysregulated nutrient sensing, stem cell deficiency, and intercellular communication alterations (Hou et al., 2019). Alzheimer's disease is marked by gradual degradation of cognitive abilities, memory loss, and behavioral disturbances. Again, Bradykinesia, akinesia, tremors, and balance disorders are the general signs of Parkinson's disease. However, both diseases can limit an individual's proper social functioning capacity (Przybylska, 2020).

The beneficial role of lycopene on aging‐related neurodegenerative disorders, such as Alzheimer's disease and Parkinson's disease, has been confirmed in both experimental and clinical trials. Lycopene is a potent compound that can ameliorate the harmful effects of neurodegenerative diseases because it can pass the blood–brain barrier (Khachik et al., 2002). Lycopene also reverses the motor abnormalities of MPTP‐induced Parkinson's disease in mouse models (Prema et al., 2015). Furthermore, lycopene prevents cognitive disorders and motor abnormalities caused by 3 nitro‐propionic acids, an irreversible succinate dehydrogenase inhibitor in Huntington's disease (Sandhir et al., 2010). Furthermore, using lycopene for a long time minimizes infarction and neuro‐apoptosis in cerebral ischemia–reperfusion and reduces the stroke risk in men (Chen, Huang, et al., 2019). Lycopene exhibits neuroprotective effects through several mechanisms. It shows neuroprotection by inhibiting oxidative stress, neuroinflammation, and neuro‐apoptosis and restoring mitochondrial functions. Moreover, lycopene exhibits a neuroprotective mechanism in CNS disorders by inhibiting microglial activation (Chen, Huang, et al., 2019) or activating the AMPK, PPARγ, and PI3K/Akt signaling (Lin et al., 2014).

A significant number of pre‐clinical trials demonstrated that lycopene protected from neurological disorders by exhibiting antioxidant, anti‐inflammation, and anti‐apoptotic actions, summarized in Table 3. Different studies on the Wister mice models showed data that lycopene protected the brain through diminishing oxidative stress, restricting the release of inflammatory mediators and their functional activities, and inhibiting neurotransmitters‐metabolizing enzymes (AchE, ADA, MAO‐A, 5′‐nucleotidase, and NTPdase) activities (Janani et al., 2012; Prakash & Kumar, 2014; Ugbaja, James, et al., 2021; Ugbaja, Ugwor, et al., 2021; Yin et al., 2014), demonstrated in Table 3. Similar findings were observed in Sprague–Dawley rats, whereas these studies represented a few additional outcomes, including upregulation of antiapoptotic Bcl2 levels and downregulating apoptotic protein cleaved caspase 3 expressions (Fu et al., 2020; Hu et al., 2017; Wu et al., 2015; Yang et al., 2018; Zhao et al., 2018). In vitro studies on SH‐SY5Y cells also demonstrated that lycopene could protect neuroblastoma cells by ameliorating oxidative damage and regulating brain apoptosis by inducing *Bcl‐2* and inhibiting *Bax*, cleaved Caspase‐3, summarized in Table 3. The evidence suggested that lycopene has higher potency in ameliorating neurological disorders. However, there is a lack of clinical evidence to support the potential of lycopene in neurological protection in humans. Therefore, more pre‐clinical and clinical studies should be done to elucidate the neuroprotective mechanisms, possible neurotoxicity, and required dose and dosage of lycopene for clinical administration.

#### 4.2.7 Kidney diseases

Attritions of well‐functioned cells from various organs and tissues are a common effect of the aging process, whereas the biological consequences of aging on the kidney include renal dysfunction and structural damage. Alterations of kidney function while normal aging occurs most frequently among all organ systems, and the glomerular filtration rate (GFR) of healthy octogenarians is only half to two‐thirds compared to younger adults (Davies & Shock, 1950; Rowe et al., 1976). Kidney aging is characterized by a loss of nephron number and size, tubulointerstitial structural changes, thickening of the glomerular basement membrane, lowering of GFR and sodium reabsorption, excessive accumulation of ECM, and increment of glomerulosclerosis (O'Sullivan et al., 2017). These phenomena are associated with acute kidney injury (AKI) and progressive chronic kidney disease (CKD). Loss of antioxidant capacity is one of the significant features of an aged kidney, which facilitates ROS formation and oxidative stress in the kidney after AKI and may increase the severity of AKI in aged kidneys (Gyurászová et al., 2020). An interplay between oxidative stress and inflammation may worsen renal complications and lead to CKD progression. It is found that ROS induces the activation of PKC, MAPK, and NF‐*κ*B pathways, which subsequently leads to the release of proinflammatory cytokines and growth factors, resulting in the development of diabetic nephropathy as well as proteinuria (Guo et al., 2020). Oxidative stress also leads to renal fibrosis by increasing TGF‐*β*1 expression, stimulating SMAD signaling, and upregulating the expression of collagen I, III, IV, fibronectin, and PAI‐1. ROS also leads to interstitial fibrosis in the kidney through activating STAT, NF‐κB, and AP‐1 intracellular signaling cascades (Ratliff et al., 2016). Targeting the antioxidant system of the kidney could be a possible way of preventing aging in kidneys and associated renal complications (Lee et al., 2019; Uddin et al., 2021).

Numerous studies have demonstrated the potential of lycopene on several renal disorders, as shown in Table 3. Lycopene has been substantiated to ameliorate renal functions and kidney tissue disorders by increasing oxidative status due to its potent antioxidant characteristics. In contrast, it contrasts medium‐inducing oxidative stresses, inflammations, auto‐phagocytosis, and apoptosis in the kidney of mouse models (Buyuklu et al., 2015). Several pre‐clinical studies reported that lycopene treatment significantly reduced serum urea and serum creatinine, as well as reversed various toxic chemical‐induced nephrotoxicity and oxidative damage by exhibiting excellent antioxidative properties. For instance, studies on different Wister rat models demonstrated that lycopene could protect the kidney from nephrotoxicity and oxidative damage by lowering serum creatinine (SCr) and blood urea nitrogen (BUN), increasing antioxidant enzymes (GSH, GST, CAT, GSH‐Px, SOD) levels, reducing MDA levels, increasing Bcl‐2 protein levels, and downregulating Bax protein levels (see Table 3) (Dogukan et al., 2011; Erman et al., 2014; Kaya et al., 2015; Li et al., 2014; Shalaby & El Shaer, 2019; Yilmaz et al., 2018). In addition, it was found that lycopene also significantly prevented necrosis, degeneration, dilation, and vacuolization in renal tubules (Kaya et al., 2015). Similar findings were also found in a few pre‐clinical trials on SD rats, whereas it is observed that lycopene attenuated nephrotoxicity and oxidative damage by improving antioxidant enzymes (CAT, GPx1, GSH, and GSH‐Px) levels, reducing MDA and TBARs levels. These studies also reported that lycopene could prevent necrosis, degeneration, dilation, vacuolization in renal tubules, interstitial edema, inter‐tubular fibrosis, focal subendocardial fibrosis, perinuclear vacuolization, and inhibited luminal cast formation, basement membrane thickening (Ateşşahin et al., 2007; Palabiyik et al., 2013; Yilmaz et al., 2006). In addition, studies on Kunming mice showed that lycopene could ameliorate nephrotoxicity and renal oxidative damage by lowering SCr, and BUN, improving antioxidant capacity, activating Nrf2/HO‐1 pathway, and inhibiting inflammatory NF‐κB mRNA expressions and pAMPK/AMPK pathway (Dai et al., 2015; Lin et al., 2018; Yu et al., 2018), summarized in Table 3. Moreover, a clinical study also demonstrated that lycopene pre‐treatment protected from nephrotoxicity, improved GFR, and reduced BUN levels in patients with cancer (Mahmoodnia et al., 2017). The wide range of nephroprotective mechanisms of lycopene validated its prospect in managing renal pathologies. In addition, more clinical studies are required to determine the dose and dosage of lycopene for kidney patients.

## 5 ROLE OF LYCOPENE AS CALORIE RESTRICTION MIMIC

Calorie restriction (CR) is defined as reducing energy intake below the needed amount to maintain weight while maintaining essential nutrient levels. CR can then be described as reducing calorie intake without malnutrition (López‐Lluch & Navas, 2020). CR facilitates preventing and postponing the onset of various diseases, including cancer, cardiovascular disease, and degenerative diseases (Guijas et al., 2020). In animals, CR decreases metabolic rate and oxidative stresses, increases insulin sensitivities, and modifies the autonomic and neuro‐endocrine nervous systems (Heilbronn & Ravussin, 2003). Reduction of metabolic rate through CR may result in decreased oxygen consumption, which may cause decreased ROS formation and possibly increased lifespan. In obese individuals (diabetic and non‐diabetic alike), CR and the resulting weight loss remarkably increase glucose metabolic rate by ameliorating the actions of insulin (Heilbronn & Ravussin, 2003). CR can also increase HDL2b level (Lane et al., 1999) and reduce several inflammatory markers (Trepanowski et al., 2011), including TNF‐α, IL 6, CRP, and NF‐қB. Apart from these, SIRT3 appears to be a critical member of the sirtuins family in the CR‐induced metabolic responses (López‐Lluch & Navas, 2020). A study demonstrated that SIRT3 levels could increase skeletal muscles by following CR and decrease by following a high‐fat diet, suggesting that sirtuins are major regulators of calorie intake and should be included in the responses (Palacios et al., 2009). However, gene expression profiling by DNA microarray demonstrated that aging is linked with multiple alterations in the gene expressions of skeletal muscles in rodents (Masoro, 1988), brains (Lee et al., 2000), and hearts (Lee et al., 2002), whereas CR can inhibit many of the occurred alterations. Park et al. (2009) used DNA microarrays to characterize aging‐related tissue‐specific gene expression profiles. They evaluated the ability of various dietary antioxidants, such as lycopene, resveratrol, acetyl‐l‐carnitine, tempol, α‐lipoic acid, and coenzyme Q_10_, to prevent the transcriptional genes of aging. The study found lycopene was effective as CR mimics in the heart, which prevented transcriptional activation of the aging‐related genes, and delayed aging processes.

A study on tomato powders or lycopene consumption and energy or calorie restrictions has been conducted to determine the effects of prostate carcinoma in rat models. The scientists investigated the efficacy of 20% dietary energy restriction on the risk of lethality by prostate carcinoma. They included 194 male rats in their study, treating them with N‐methyl‐N‐nitrosourea and testosterone for the induction of prostate carcinoma, and fed them whole tomato powders that contained 13 mg lycopene per kg. This study demonstrated that the restrictions in energy intake might cause a 32% abatement in prostate carcinoma‐related death if rats are fed tomato powders or lycopene (Boileau et al., 2003; Gann & Khachik, 2003).

Hepatic steatosis or fatty liver disease is another severe complication, generally resulting from being overweight in childhood. Calorie‐restricted regimen (CRR) has been substantiated as one of the effective therapies for this complication. A recent study has evaluated the effectiveness of lycopene‐rich tomato sauce combined with oreganos and basils against hepatic steatosis in obese children conducted on CRR. A randomized cross‐over clinical trial was procured for 60 days on 61 obese children, and the children were assigned either to CRR (27 children) or CRR along with lycopene‐rich tomato juice supplements (34 children). The study demonstrated that the consuming tomato supplements group reduced body mass index (BMI), homeostatic model assessment for insulin resistance (HOMA‐IR) value, cholesterol, TG, hepatic measurement, and steatosis condition CRR. In addition, tomato juice supplements on CRR improved glucose and lipids metabolism, ameliorated oxidation and inflammation conditions, and modulated mitochondria's metabolism of T lymphocytes. These all contributed to the balanced immunity of the impaired children due to CRR. Though CRR generally impairs glycolysis and growth of T lymphocytes, lycopene in tomato juice supplements can activate T lymphocyte‐mediated glycolytic metabolism. This experiment proved that the lycopene of tomato juice on CRR plays a pivotal role in providing protection and prevention support to obese children (Negri et al., 2020).

## 6 PROSPECTS AND LIMITATIONS

The prospects of lycopene are not only limited to its anti‐aging properties, but also the food industry can be benefitted from high‐quality lycopene products that comply with food safety regulations. The new dietary recommendation to increase the intake of antioxidant‐rich fruits and vegetables has sparked interest in lycopene's involvement in disease prevention (Palozza et al., 2012). In addition, lycopene is a plant pigment used as a cosmetic colorant. Lycopene is naturally red and suitable for lipstick, blush, and many other cosmetics. Moreover, lycopene is now commercially available as LycoMato and LycoRed to treat hypertension and oral leukoplakia, respectively (Misra et al., 2006; Rao & Shen, 2002).

Despite numerous prospects for lycopene, it has some limitations. A recent study demonstrated that the structural localization of lycopene in the chloroplast of fruits and vegetables is considered a crucial factor in limiting the bioavailability of lycopene by dietary intake because chloroplast shows more excellent resistance against gastric and gastrointestinal digestion (Schweiggert et al., 2014). Therefore, structures of the food matrix can greatly predetermine the bioavailability of lycopene (Petyaev, 2016). Its use can make someone more vulnerable to bleeding during and after surgery. Lycopene supplementation during pregnancy is unhealthy. High intakes of lycopene‐containing foods have been linked to lycopenemia, marked by orange skin discoloration. Excessive lycopene consumption has been linked to various gastrointestinal problems, including diarrhea, nausea, stomach cramps, gas, and vomiting (Trumbo, 2005). However, considering the wide range of functional roles and minimum adverse effects of lycopene, it has been prospected that lycopene can be a potential phytomedicine for delaying aging degradation and managing aging‐related chronic disorders.

## 7 CONCLUSION

Deaths related to aging and aging‐related disorders are increasing day by day. Therefore, alternative approaches have become crucial to public health to keep the disorders under control by considering the side effects of traditional medications and uncontrollable disease conditions. Our review discussed its chemistry and biochemistry, evaluated its potential against aging biomarkers and aging‐related disorders, and indicated its limitations with possible solutions. However, further extensive research on this therapeutic agent is required to unravel its role in gene expressions, determine the precise doses in physiological rather than pharmacological conditions, and comprehend its genotoxic and teratogenic effects. Furthermore, some discrepancies regarding lycopene's metabolites type in tissues, their absorbance efficiency, and their bioactivities in physiological concentration need to be resolved. Also, it needs to be investigated whether lycopene directly or its metabolites manifest anti‐aging activities.

## AUTHOR CONTRIBUTIONS

**Mehedy Hasan Abir:** Visualization (equal); writing – original draft (lead). **A. G. M. Sofi Uddin Mahamud:** Conceptualization (lead); validation (equal); visualization (lead); writing – review and editing (supporting). **Sadia Haque Tonny:** Writing – original draft (equal). **Mithila Saha Anu:** Writing – original draft (equal). **K. H. Sabbir Hossain:** Writing – original draft (equal). **Ismam Ahmed Protic:** Writing – original draft (equal). **Md Shihab Uddine Khan:** Writing – original draft (supporting). **Artho Baroi:** Writing – original draft (supporting). **Akhi Moni:** Validation (equal); writing – review and editing (equal). **Md Jamal Uddin:** Conceptualization (equal); project administration (equal); supervision (lead); validation (lead); writing – review and editing (equal).

## CONFLICT OF INTEREST STATEMENT

The authors report no competing interests to declare.

## ETHICS STATEMENT

This study does not involve any human or animal testing.

## Data Availability

Data sharing is not applicable as no new data were generated or analyzed during this study.
